# Supplementary material for: A hierarchically assembled 88-nuclei silver-thiacalix[4]arene nanocluster
Source: Nat Commun. 2020 Jan 16;11:308. doi: 10.1038/s41467-019-13682-5 (PMC6965622; doi:10.1038/s41467-019-13682-5)
Supplement: Supplementary file 1 — Supplementary information [file 41467_2019_13682_MOESM1_ESM.pdf]

Supplementary Information

A Hierarchically Assembled 88-Nuclei Silver-Thiacalix[4]arene Nanocluster

Wang et al

Supplementary Methods.

Materials and reagents

The $(EtSAg)_n$[1] and *p-tert*-butylthiacalix[4]arene (H_4TC4A)[2] were prepared by following the reported procedure. EtSH (Adamas-beta®) and AgOAc (Adamas-beta®) were purchased from Shanghai Titan Scientific Co.,Ltd. All other chemicals and solvents used in the syntheses were of analytical grade and used without further purification.

Elemental analysis

The elemental analyses (C, H, N contents) were determined on a Vario EL III analyzer.

Fourier transform infrared spectroscopy (FTIR)

FTIR spectra were recorded on a Bruker Tensor II spectrophotometer (Bruker Optics GmbH, Ettlingen, Germany) utilizing a single attenuated total reflectance (ATR) accessory covering a wavenumber range from 400 to 4000 cm^{-1}. The final spectrum was the average of 32 scans accumulated using Bruker's Opus software 8.1, taken at 4 cm^{-1} resolution. The samples were measured under the same mechanical force pushing the samples in contact with the diamond window.

UV/Vis spectroscopy

The diffuse-reflectance spectra were performed on UV−Vis spectrophotometer (Evolution 220, ISA-220 accessory, Thermo Scientific) using a built-in 10 mm silicon photodiode with a 60 mm Spectralon sphere.

Transmission electron microscopy (TEM)

High-resolution TEM images were recorded on a FEI TALOS F200 transmission electron microscope operating at 200 kV. Several crystals of **SD/Ag88a** were dissolved in dichloromethane or dispersed in ethanol under the ultrasound condition. The solution or emulsion was dropped onto the ultrathin carbon film with a pipette, and then left to dry and observed under a transmission electron microscope.

Dynamic light scattering (DLS)

The diameters of **SD/Ag88a** were measured by DLS using a Zetasizer Nano ZS90 (Malvern Instruments, UK). The measurements were conducted at a scattering angle

of 90° at 25 °C. The measurements were repeated thrice for each sample.

Elemental mapping images

Morphology of the samples and elemental composition analyses were measured using an SU-8010 field emission scanning electron microscope (FESEM; Hitachi Ltd., Tokyo, Japan) equipped with an Oxford-Horiba Inca XMax50 energy dispersive X-ray spectroscopy (EDS) attachment (Oxford Instruments Analytical, High Wycombe, England).

Thermogravimetic analysis (TGA)

Thermogravimetic analysis (TGA) was done in a TA SDT Q600 thermal analyzer at a heating rate of 20°C/min under N_2 atmosphere (200 mL/min) from 20 to 800 °C.

Powder X-ray diffraction (PXRD)

Powder X-ray diffraction (PXRD) analyses were carried out on a microcrystalline powder using a Rigaku Oxford Diffraction XtaLAB Synergy-S diffractometer using Cu radiation (λ = 1.54184 Å). The PXRD patterns were processed with the *CrysAlis^{Pro}* software suite[3] using the Powder function.

Mass spectra

Mass spectra were recorded on an Agilent 6224 (Agilent Technologies, USA) ESI-TOF-MS spectrometer. Sample solutions are infused by a syringe pump at 4 μL/min. Data were acquired using the following settings: ESI capillary voltage was set at 3500 V (−) ion mode and fragmentor at 200 V. The liquid nebulizer was set to 15 psig and the nitrogen drying gas was set to a flow rate of 4 L/min. Drying gas temperature was maintained at 150 °C. The data analyses of mass spectra were performed based on the isotope distribution patterns using Agilent MassHunter Workstation Data acquisition software (Version B.05.00). The reported *m/z* values represent monoisotopic mass of the most abundant peak within the isotope pattern.

Photocurrent measurement

The photocurrent test was carried out on a CHI660E electrochemistry workstation. The crystals (10 mg) of $(EtSAg)_n$, **SD/Ag88a** or **SD/Ag88b** and naphthol (0.5 wt. %, 10 μL) were dispersed in 0.5 mL ethanol, the mixture was sonicated for about 30 min. Then a 40 μL solution was transferred by pipet dropped on the cleaned ITO glass and

the coated film was obtained after evaporation under ambient atmosphere. The prepared ITO glass film was used as working electrode, a Pt wire as the counter electrode, and an Ag/AgCl electrode as the reference electrode. A Na_2SO_4 aqueous solution (0.2 M) was used as the medium.

Supplementary Note.

Single crystals of **SD/Ag88a** and **SD/Ag88b** with appropriate dimensions were chosen under an optical microscope and quickly coated with high vacuum grease (Dow Corning Corporation) to prevent decomposition. Single-crystal X-ray diffraction data of **SD/Ag88a** and **SD/Ag88b** were collected on a Rigaku Oxford Diffraction XtaLAB Synergy diffractometer equipped with a HyPix-6000HE area detector at 100 K using Mo Kα (λ = 0.71073 Å) and Cu Kα (λ = 1.54184 Å) from PhotonJet micro-focus X-ray Source. The diffraction images were processed and scaled using the *CrysAlis^Pro^* software suite.[3] The structure was solved using the charge-flipping algorithm, as implemented in the program *SUPERFLIP*[4] and refined by full-matrix least-squares techniques against F_o^2 using the SHELXL program[5] through the OLEX2 interface.[6] Hydrogen atoms at carbon were placed in calculated positions and refined isotropically by using a riding model. Appropriate restraints or constraints were applied to the geometry and the atomic displacement parameters of the atoms in the cluster. All structures were examined using the Addsym subroutine of PLATON[7] to ensure that no additional symmetry could be applied to the models. Pertinent crystallographic data collection and refinement parameters are collated in Supplementary Table 1. Selected bond lengths and angles are collated in Supplementary Table 2.

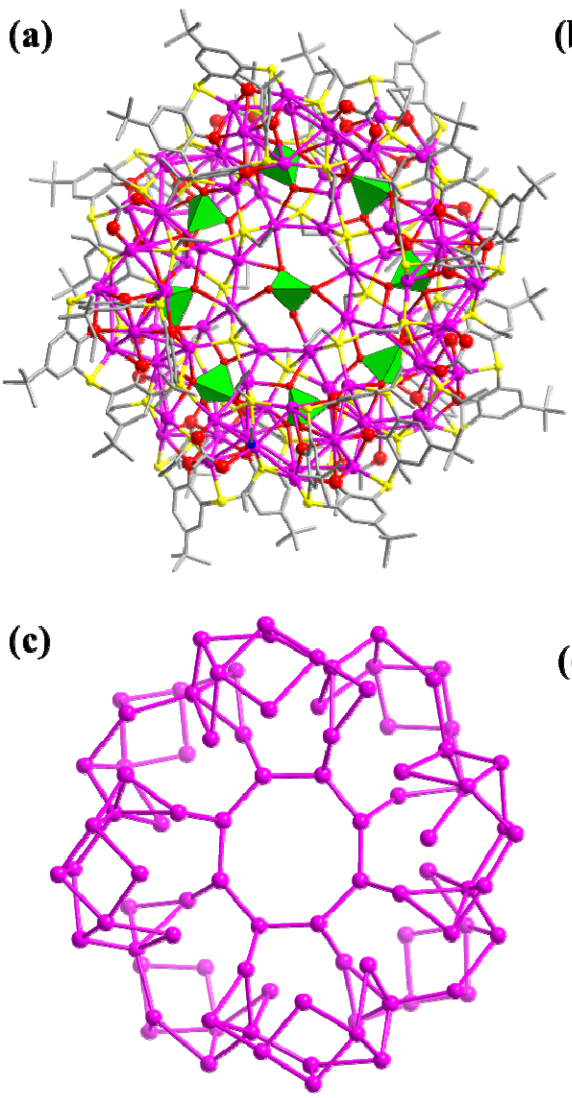

Supplementary Figure 1: Single-crystal X-ray structure of **SD/Ag88b**. (**a**) and (**b**) Total structures of **SD/Ag88b** viewed along two orthogonal directions. The compared views of the skeletal structures of **SD/Ag88b** (**c**) and **SD/Ag88a** (**d**) by removing all organic ligands and anion templates. Color labels: purple, Ag; yellow, S; gray, C; red, O; blue, N; green polyhedra, CrO_4^{2-}.

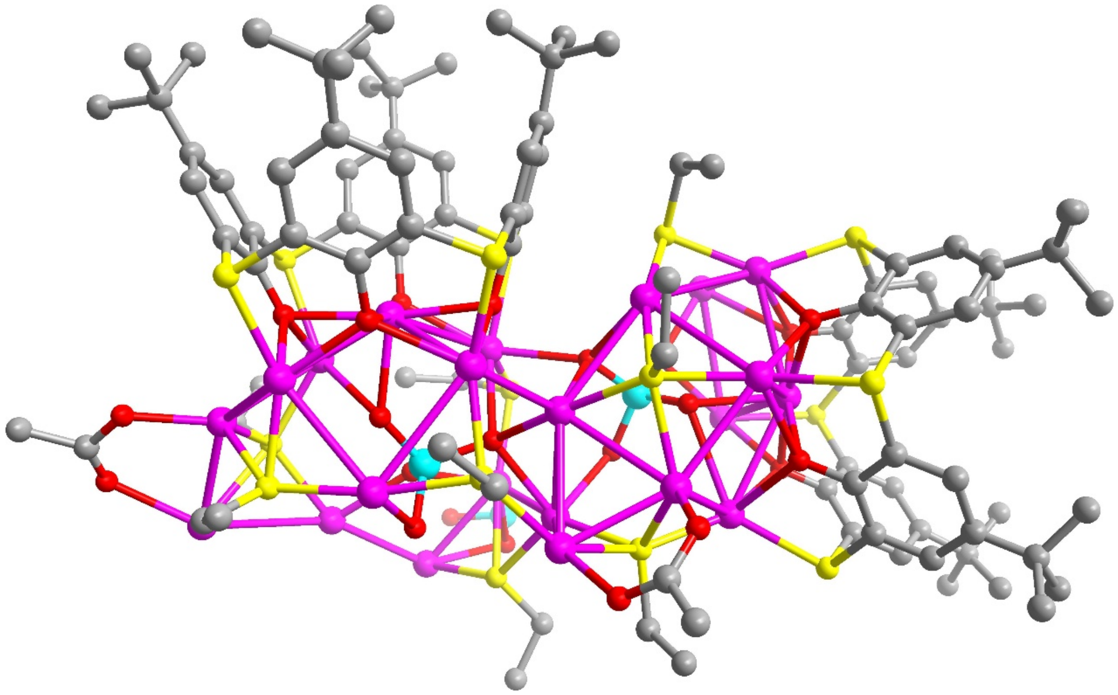

Supplementary Figure 2: The structure in an asymmetric unit of **SD/Ag88a**.

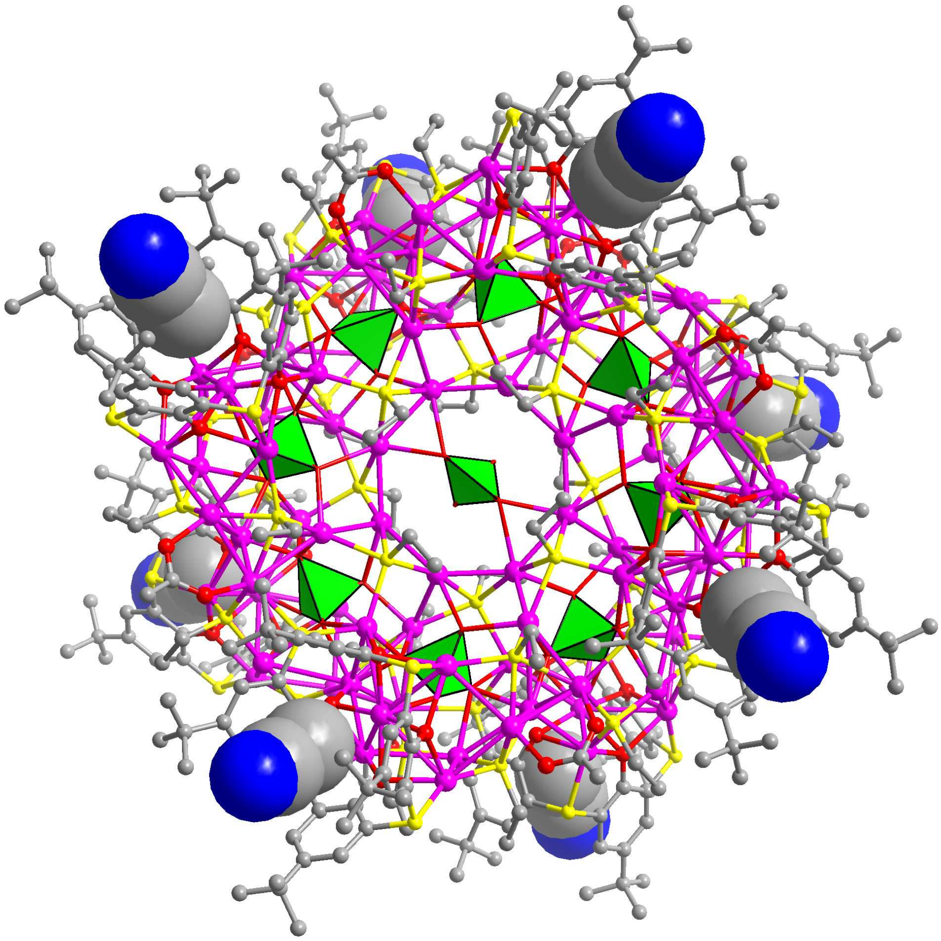

Supplementary Figure 3: The molecular structure of **SD/Ag88a** with each $TC4A^{4-}$ trapping one CH_3CN molecule.

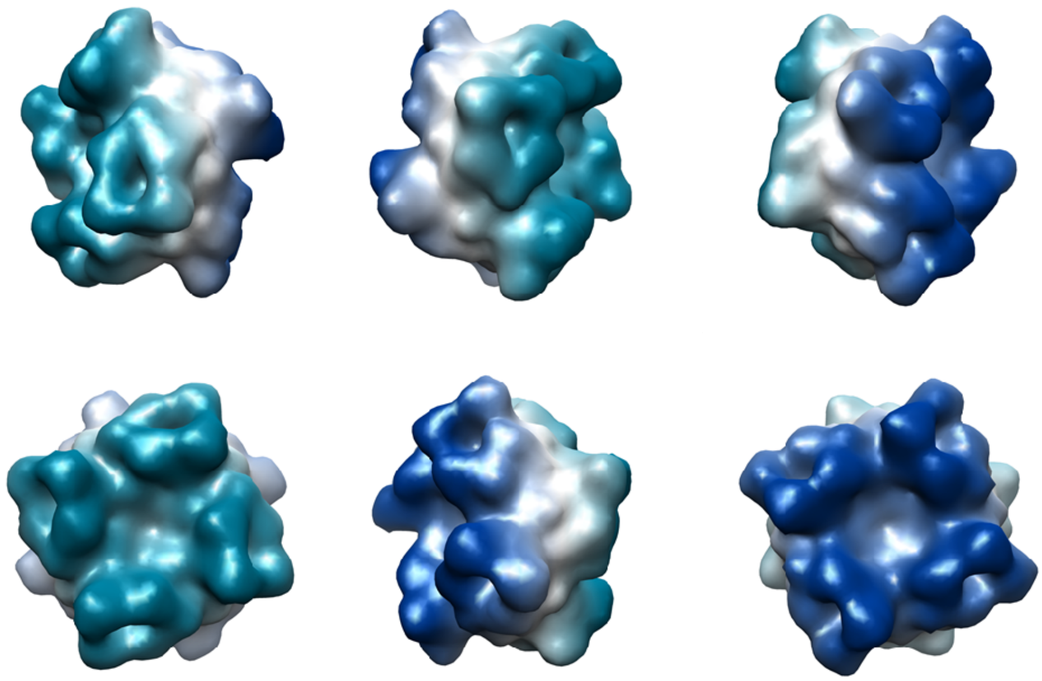

Supplementary Figure 4: The surface of **SD/Ag88a** calculated via 3V Volume Assessor program[8] by rolling a virtual probe (0.8 Å) on the surface viewed along six different orientations.

(a)

(b)

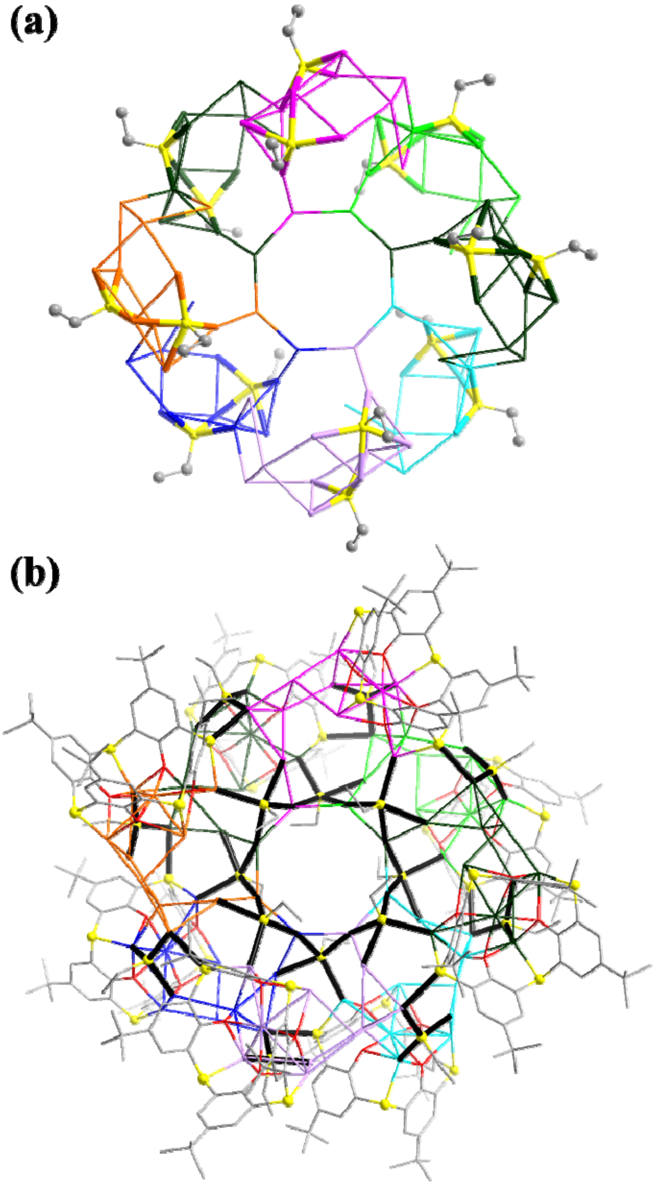

Supplementary Figure 5: The ligand distributions in each Ag_{11} SBU and between adjacent Ag_{11} SBUs. (**a**) The μ_4-EtS^- ligands capping on each Ag_{11} SBU. (**b**) The EtS^- and $TC4A^{4-}$ ligands linking adjacent Ag_{11} SBUs. The Ag-S bonds linking adjacent Ag_{11} SBUs are represented by black bold lines.

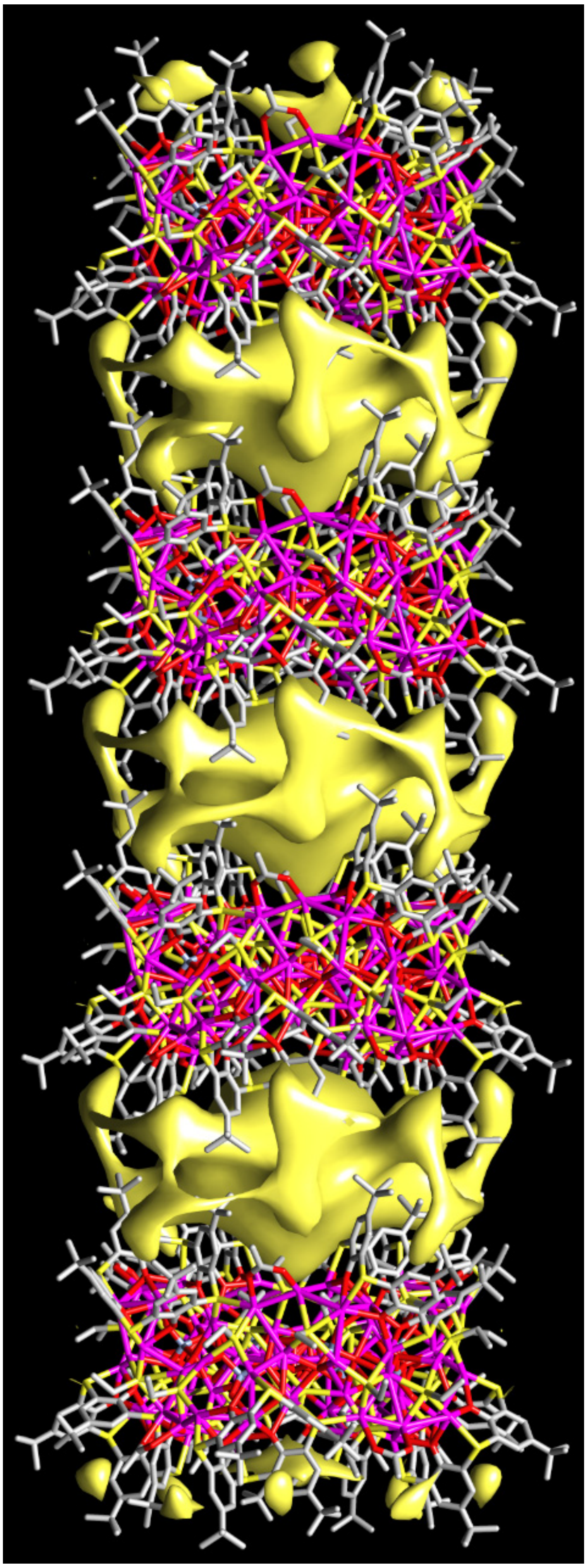

Supplementary Figure 6: The solvent-accessible voids of **SD/Ag88a** packed into one-dimensional column calculated via 3V Volume Assessor program.[8]

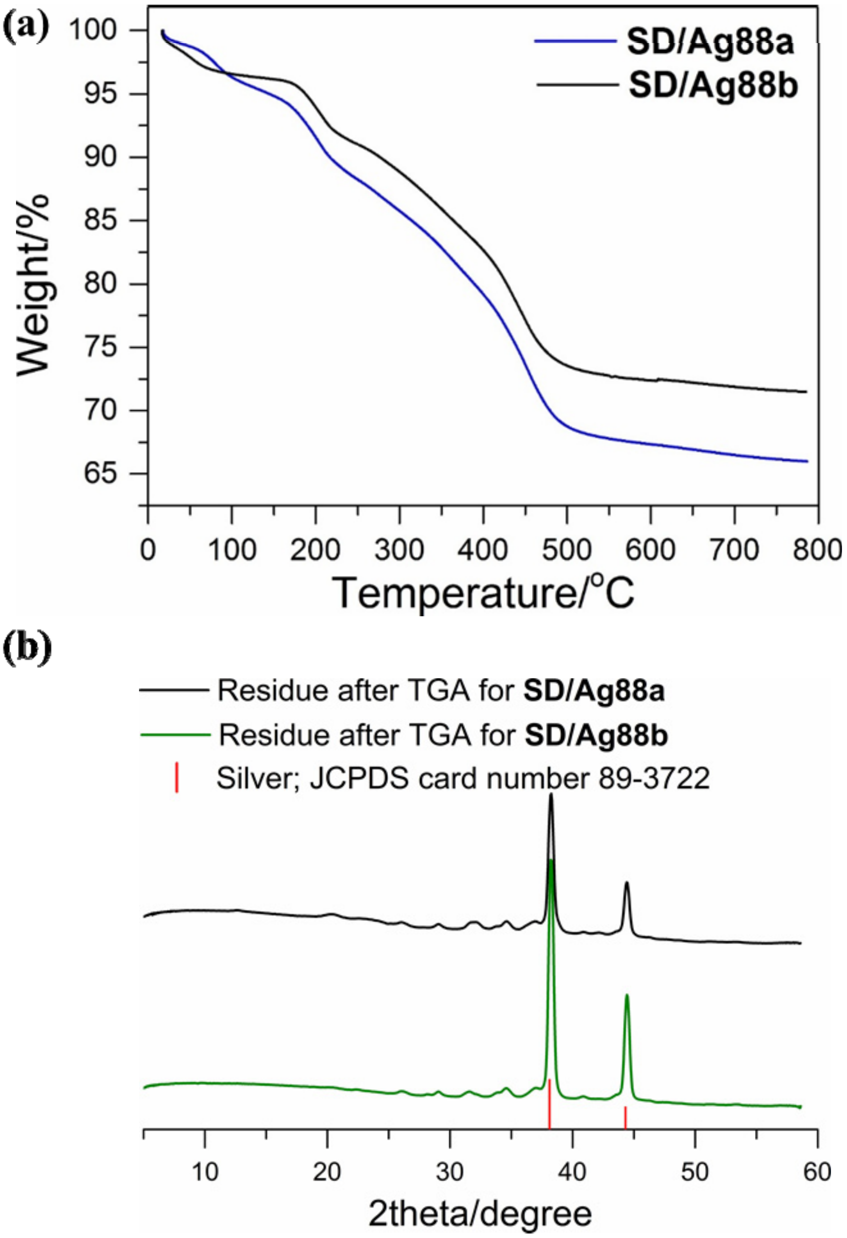

Supplementary Figure 7: (a) The TGA curves of **SD/Ag88a** and **SD/Ag88b.** (b) The PXRD patterns of residues after TG analysis for **SD/Ag88a** and **SD/Ag88b**.

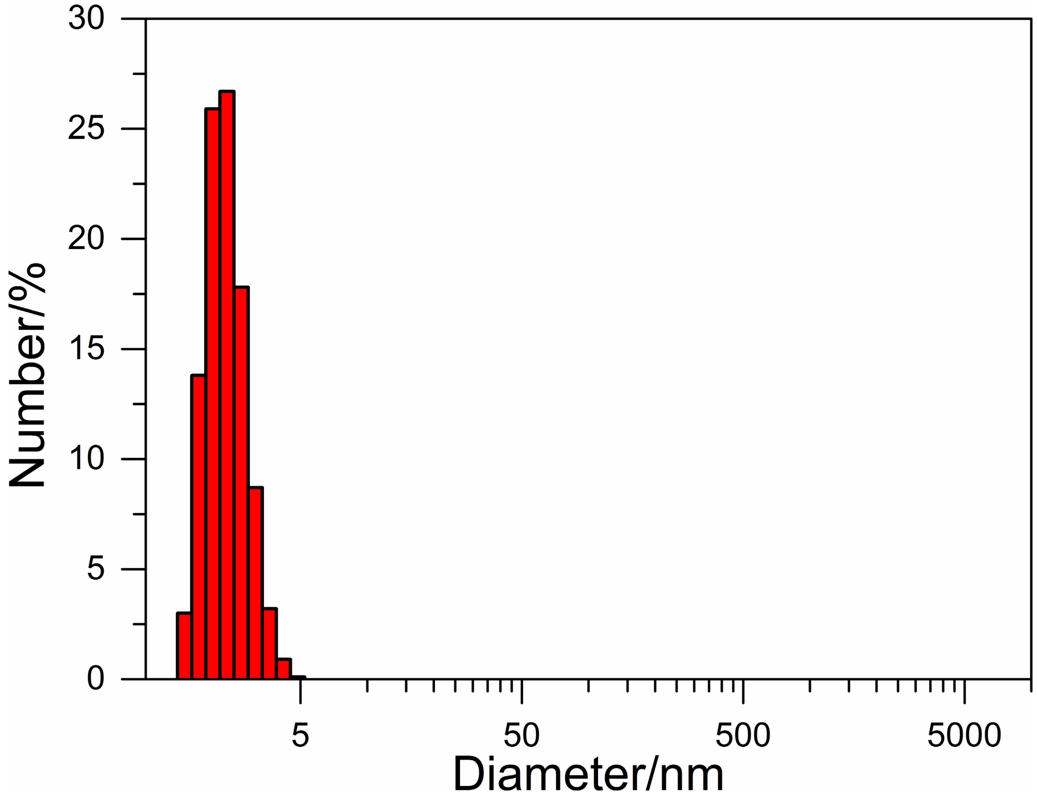

Supplementary Figure 8: Size distribution of species in a CH_2Cl_2 dilute solution of **SD/Ag88a** determined by dynamic light scattering (DLS).

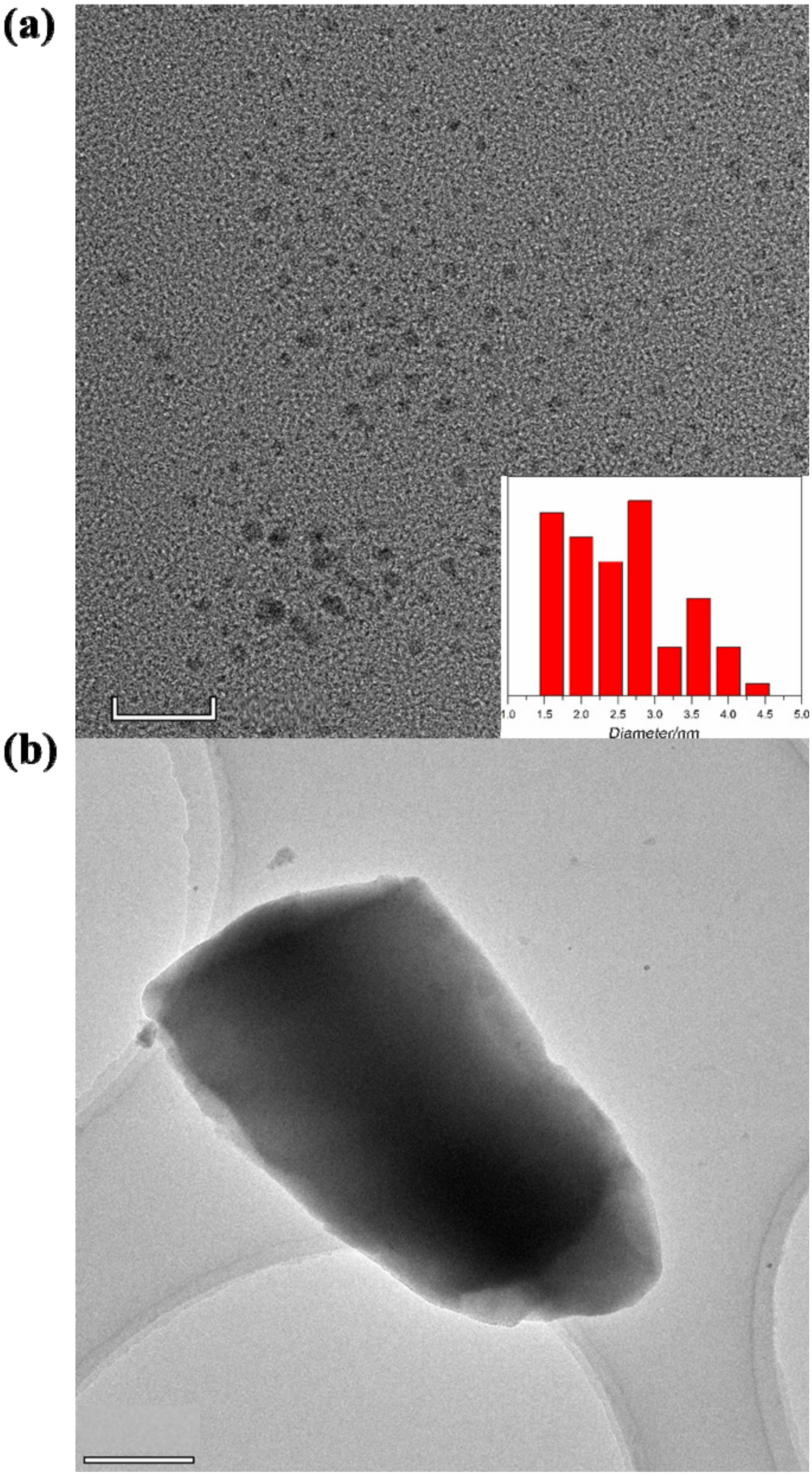

Supplementary Figure 9: TEM images of **SD/Ag88a** dissolved in CH_2Cl_2 (a) and dispersed in ethanol (b). The scale bars are 20 nm (a) and 500 nm (b).

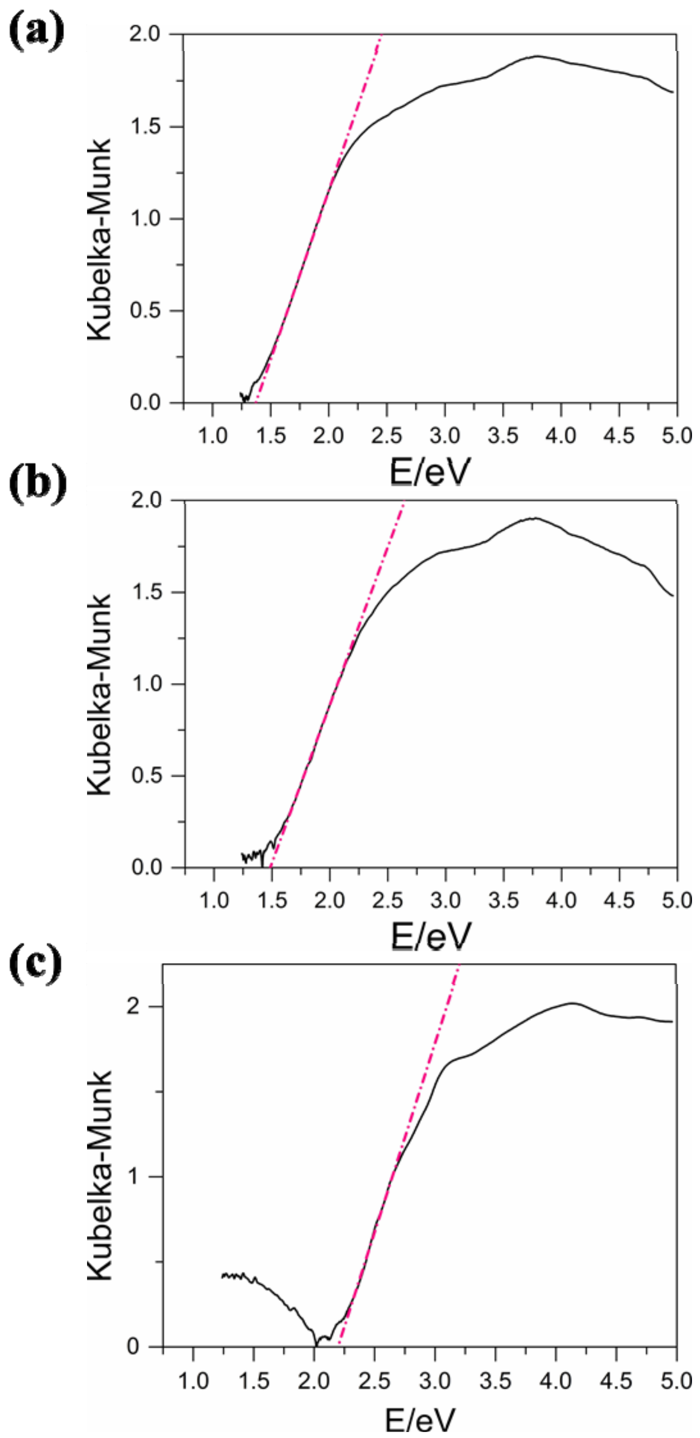

Supplementary Figure 10: Absorption spectra of **SD/Ag88a**, **SD/Ag88b** and (EtSAg)$_n$ precursor derived from the diffuse reflectance spectra through Kubelka-Munk function.

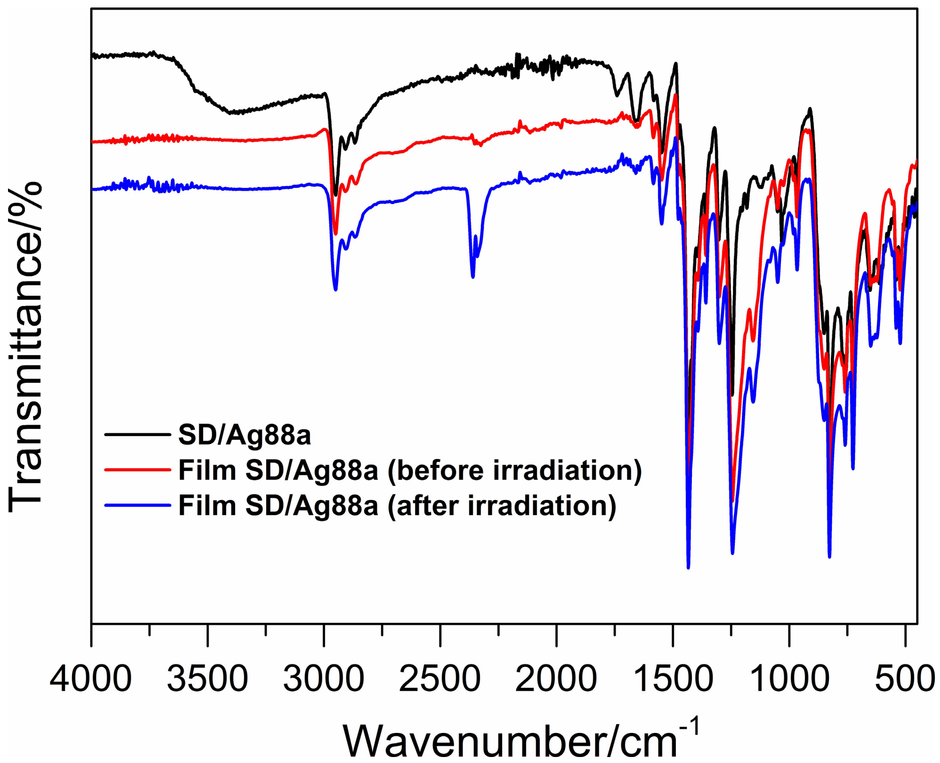

Supplementary Figure 11: The compared IR spectra of **SD/Ag88a**.

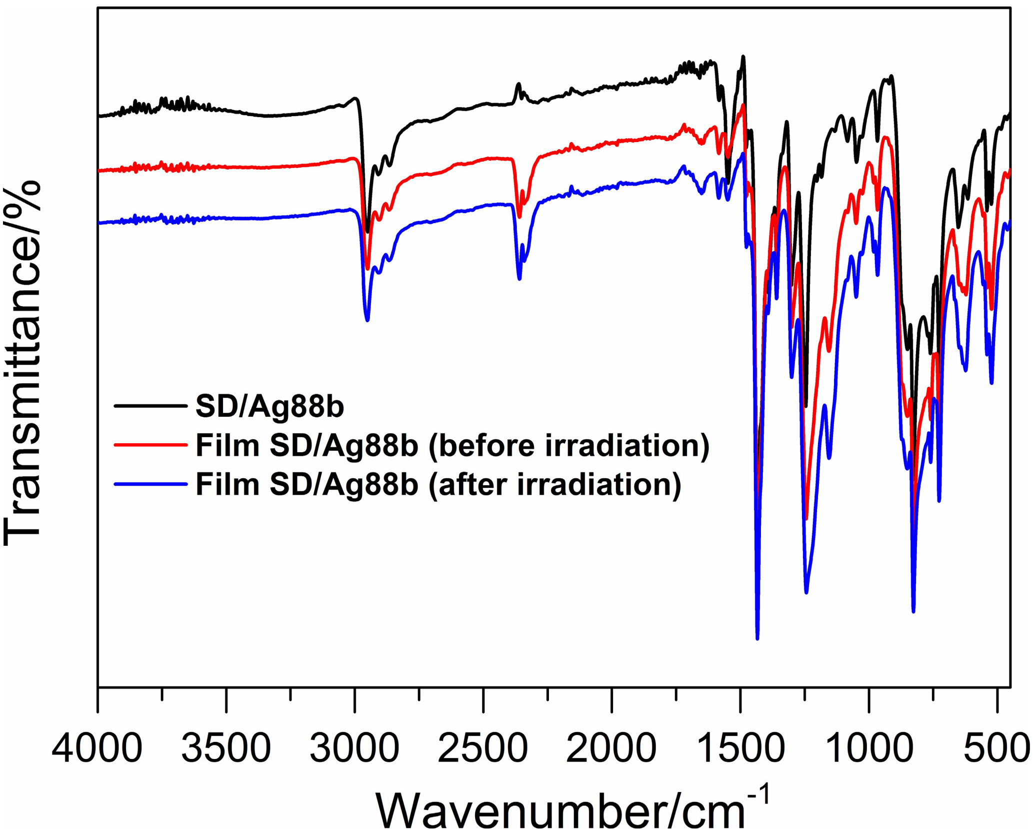

Supplementary Figure 12: The compared IR spectra of **SD/Ag88b**.

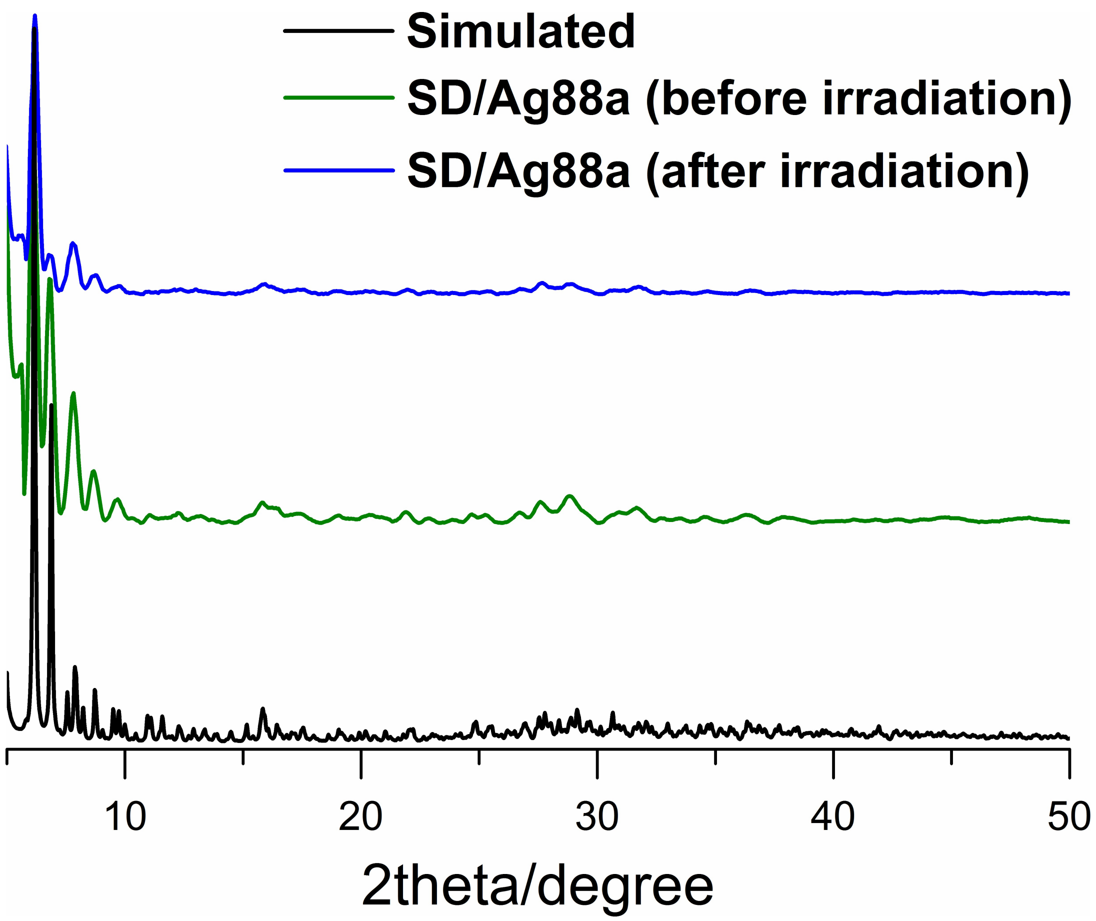

Supplementary Figure 13: The compared PXRD patterns of **SD/Ag88a** (The simulated PXRD pattern from SCXRD, experimental PXRD patterns of as-synthesized product, experimental PXRD patterns of the sample of **SD/Ag88a** after 420 nm light irradiation).

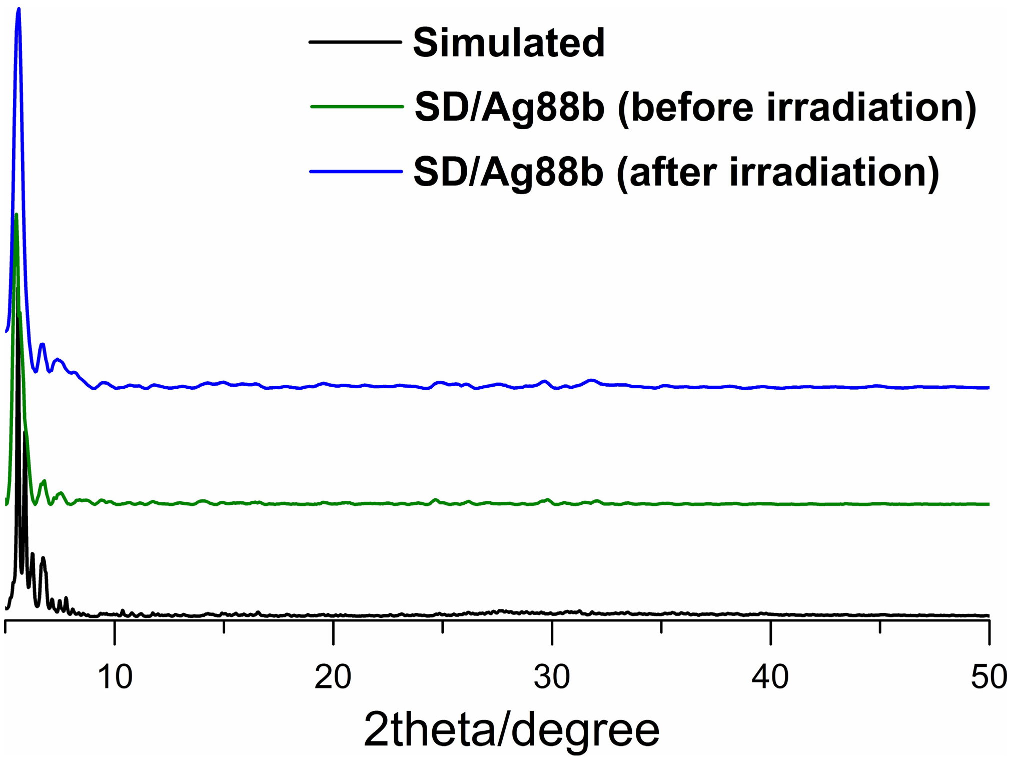

Supplementary Figure 14: The compared PXRD patterns of **SD/Ag88b** (The simulated PXRD pattern from SCXRD, experimental PXRD patterns of as-synthesized product, experimental PXRD patterns of the sample of **SD/Ag88b** after 420 nm light irradiation).

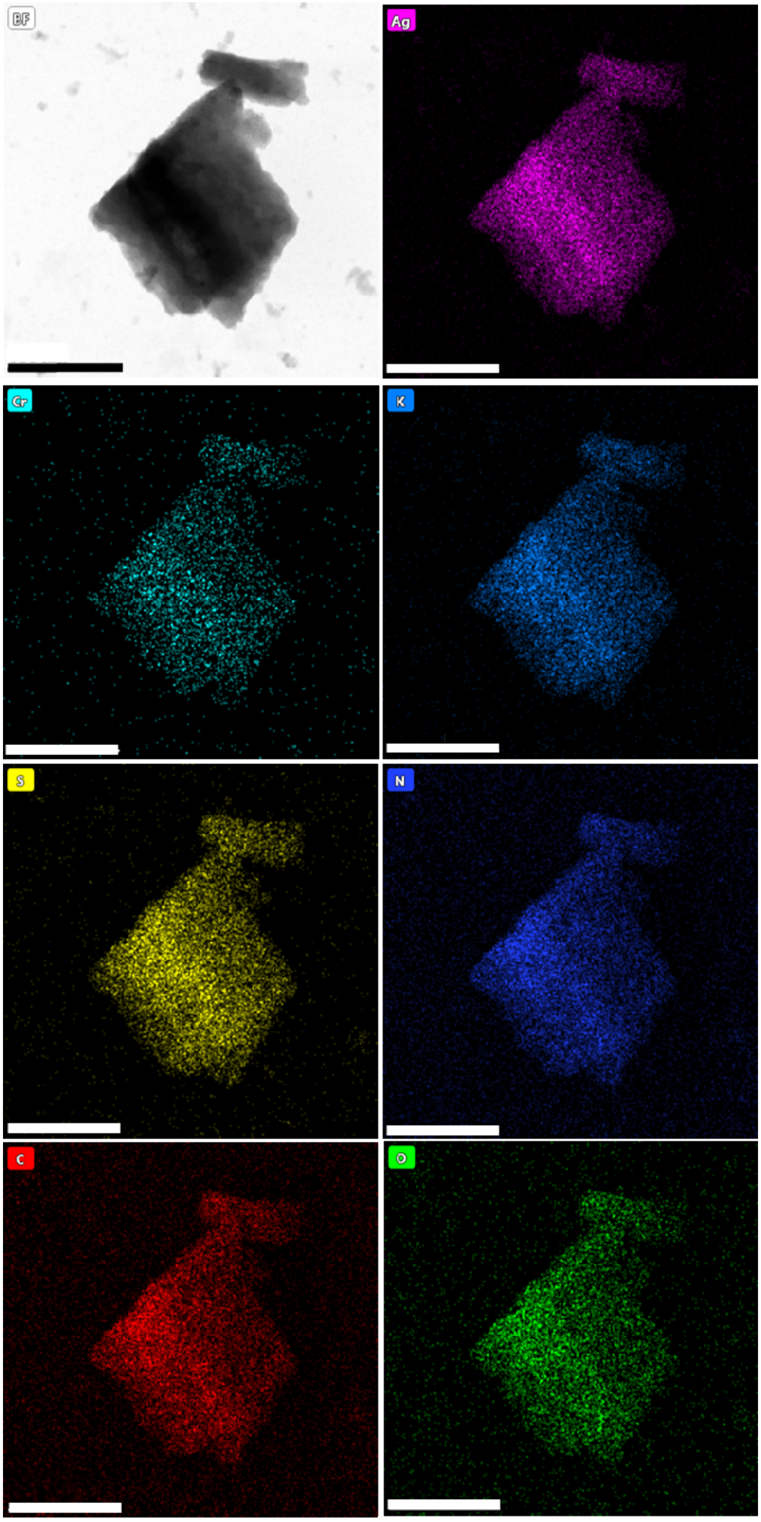

Supplementary Figure 15: SEM and elemental mapping images of **SD/Ag88a**. The scale bar is 500 nm.

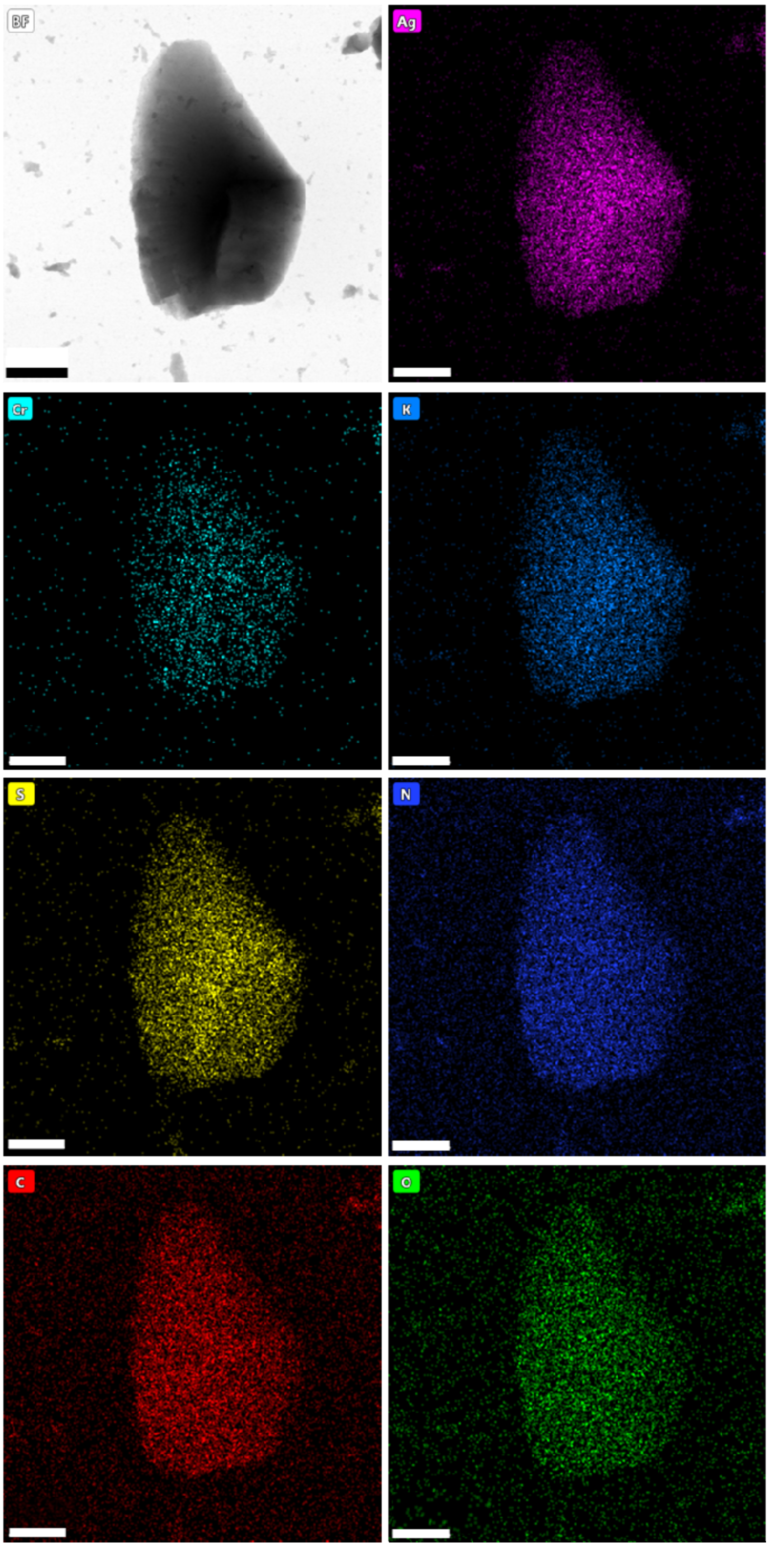

Supplementary Figure 16: SEM and elemental mapping images of **SD/Ag88b**. The scale bar is 500 nm.

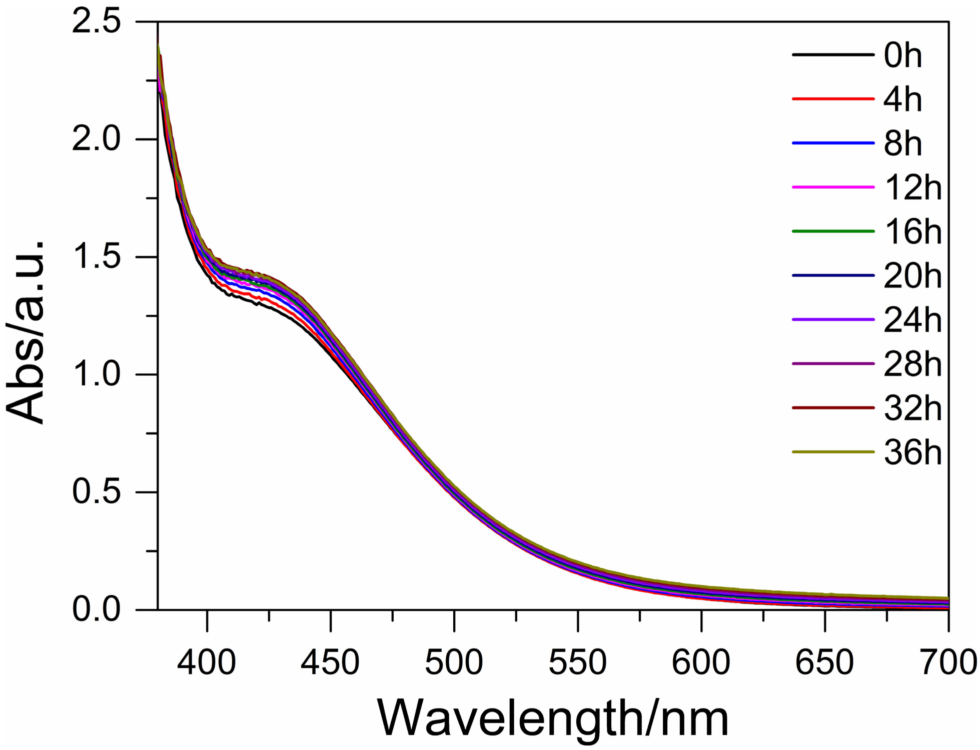

Supplementary Figure 17: Time-course UV-Vis spectra of **SD/Ag88a** in CH_2Cl_2.

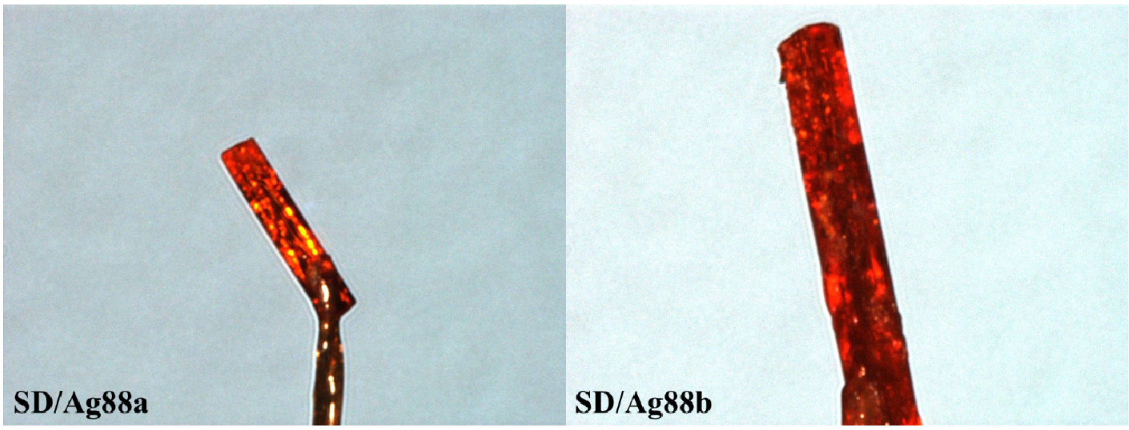

Supplementary Figure 18: Single-crystal photographs of **SD/Ag88a** and **SD/Ag88b**.

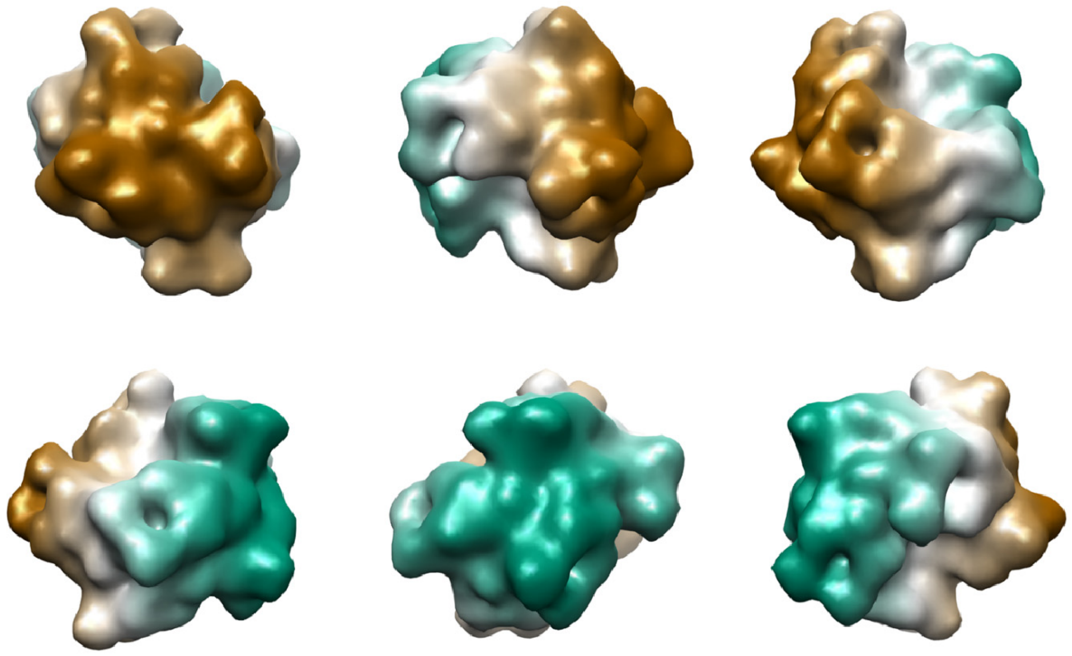

Supplementary Figure 19: The surface of **SD/Ag88b** calculated via 3V Volume Assessor program[8] by rolling a virtual probe (0.8 Å) on the surface viewed along six different orientations.

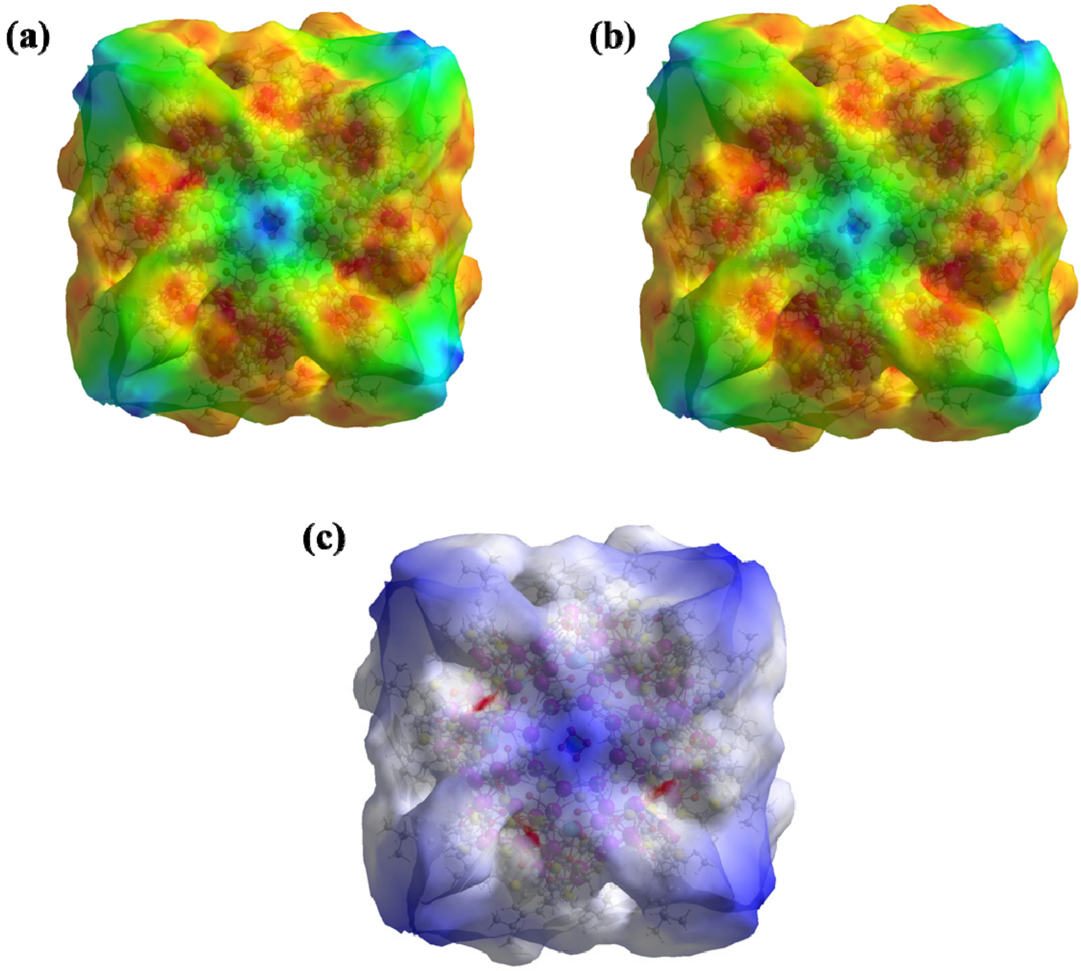

Supplementary Figure 20: Hirshfeld surface[9] of **SD/Ag88a** with d_{i} (**a**), d_{e} (**b**) d_{norm} (**c**) mapped in colour. In all cases red represents the closest contacts, and blue the most distant contacts.

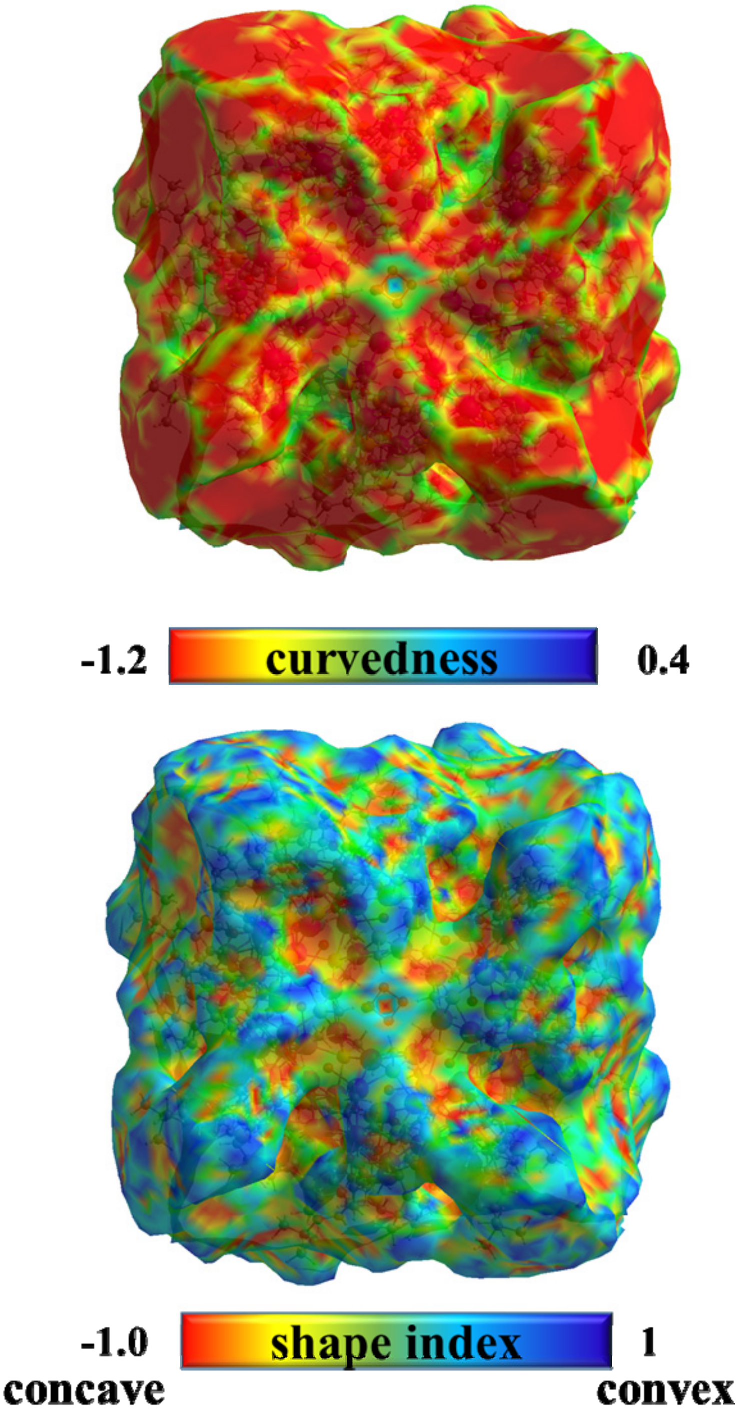

Supplementary Figure 21: Hirshfeld surface[9] of **SD/Ag88a** with curvedness and shape index mapping.

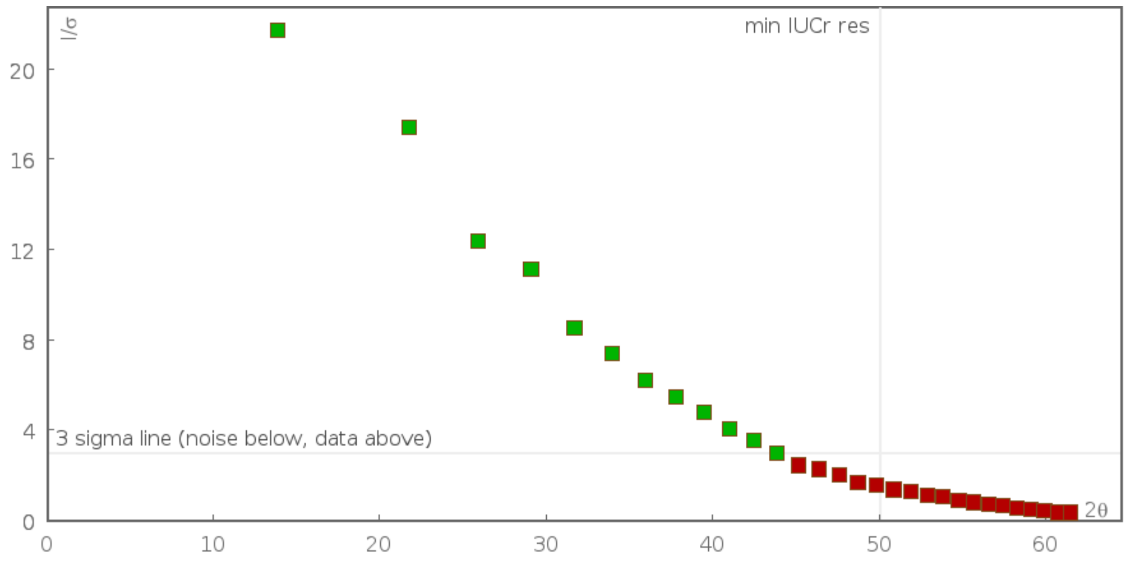

Supplementary Figure 22: The I/sigma vs. resolution plot of **SD/Ag88a** derived from reflection data statistics using OLEX 1.2.10.[6]

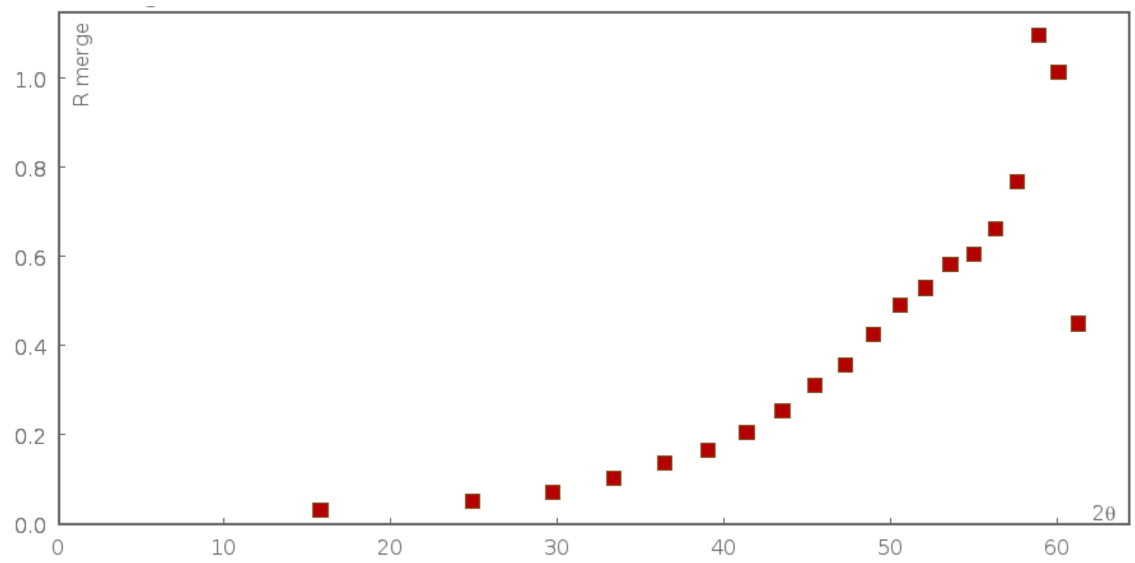

Supplementary Figure 23: The R_{merge} vs. resolution plot of **SD/Ag88a** derived from reflection data statistics using OLEX 1.2.10.[6]

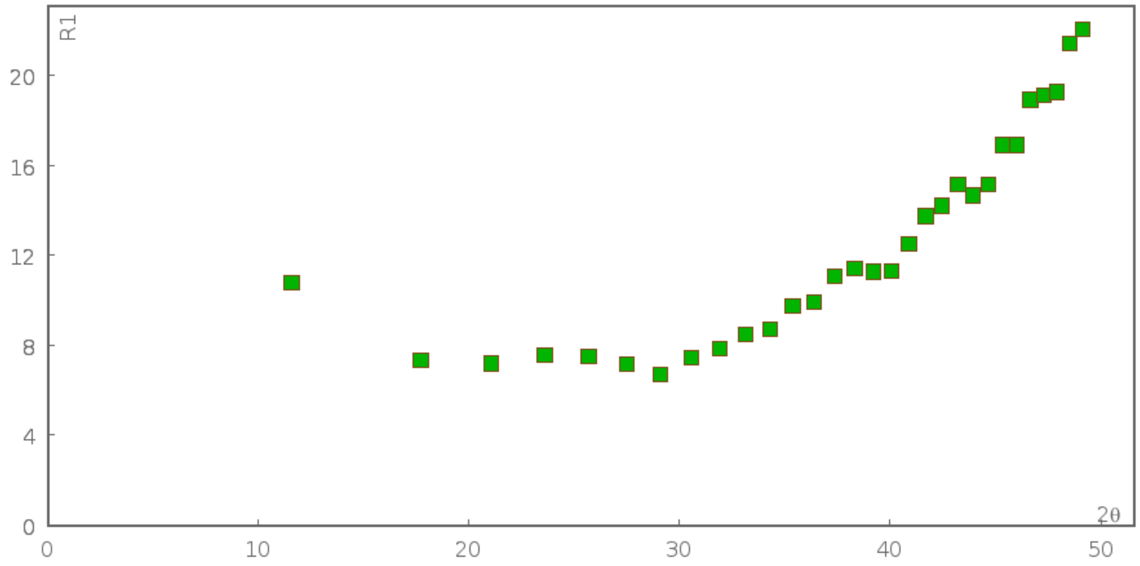

Supplementary Figure 24: The R_1 factor vs. resolution plot of **SD/Ag88a** derived from reflection data statistics using OLEX 1.2.10.[6]

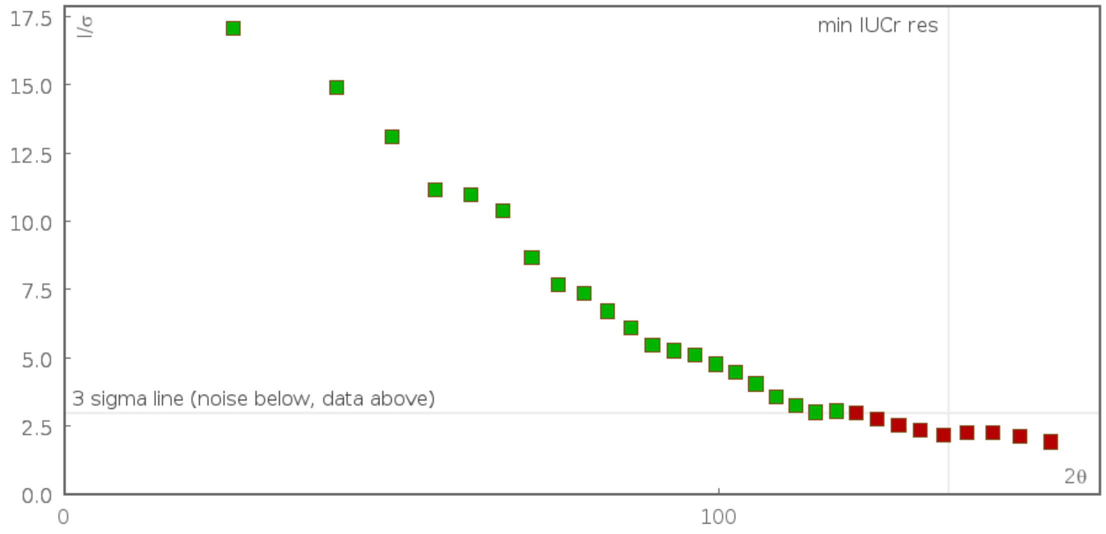

Supplementary Figure 25: The I/sigma vs. resolution plot of **SD/Ag88b** derived from reflection data statistics using OLEX 1.2.10.[6]

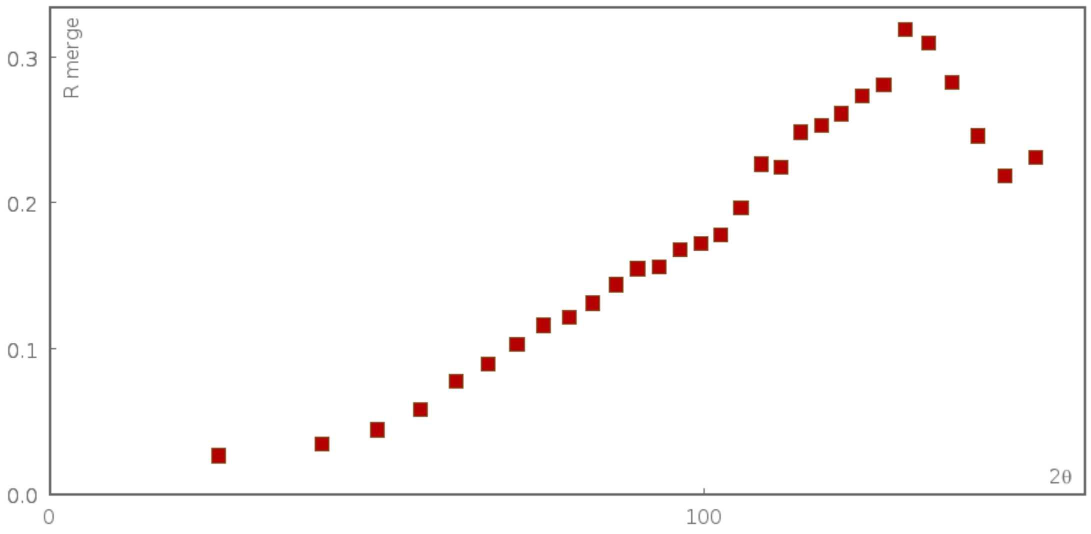

Supplementary Figure 26: The R_{merge} vs. resolution plot of **SD/Ag88b** derived from reflection data statistics using OLEX 1.2.10.[6]

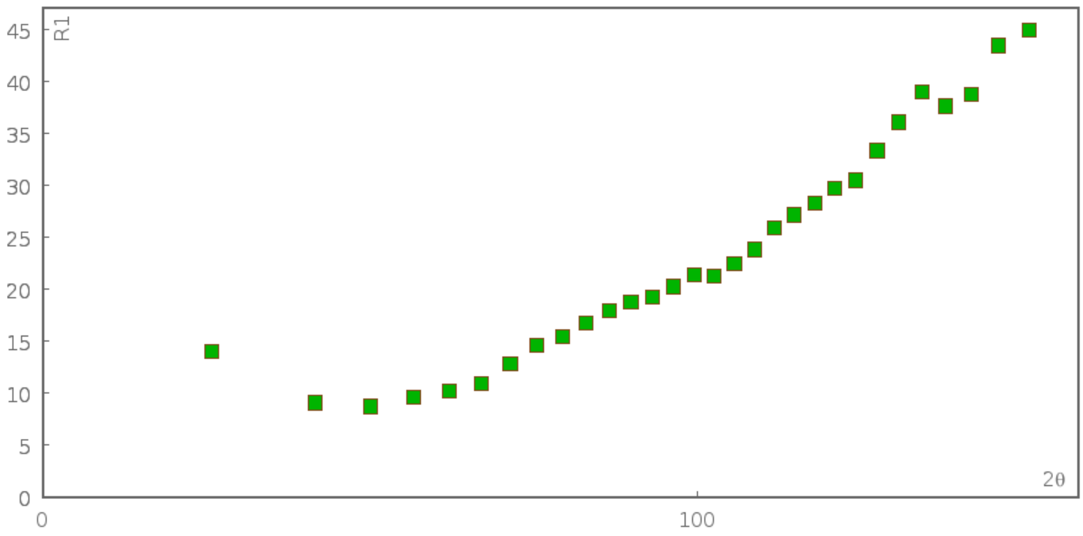

Supplementary Figure 27: The R_1 factor vs. resolution plot of **SD/Ag88b** derived from reflection data statistics using OLEX 1.2.10.[6]

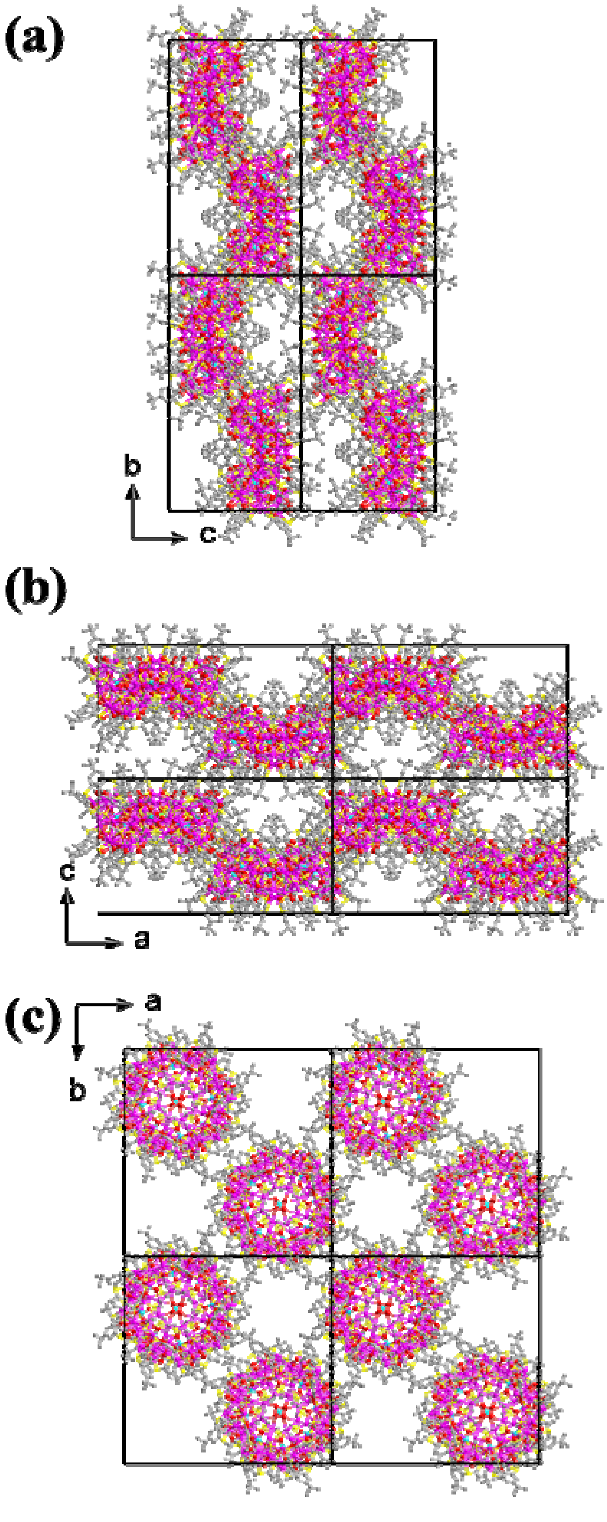

Supplementary Figure 28: Molecules packing diagrams in 2×2×2 unit cells of **SD/Ag88a** viewed from different directions.

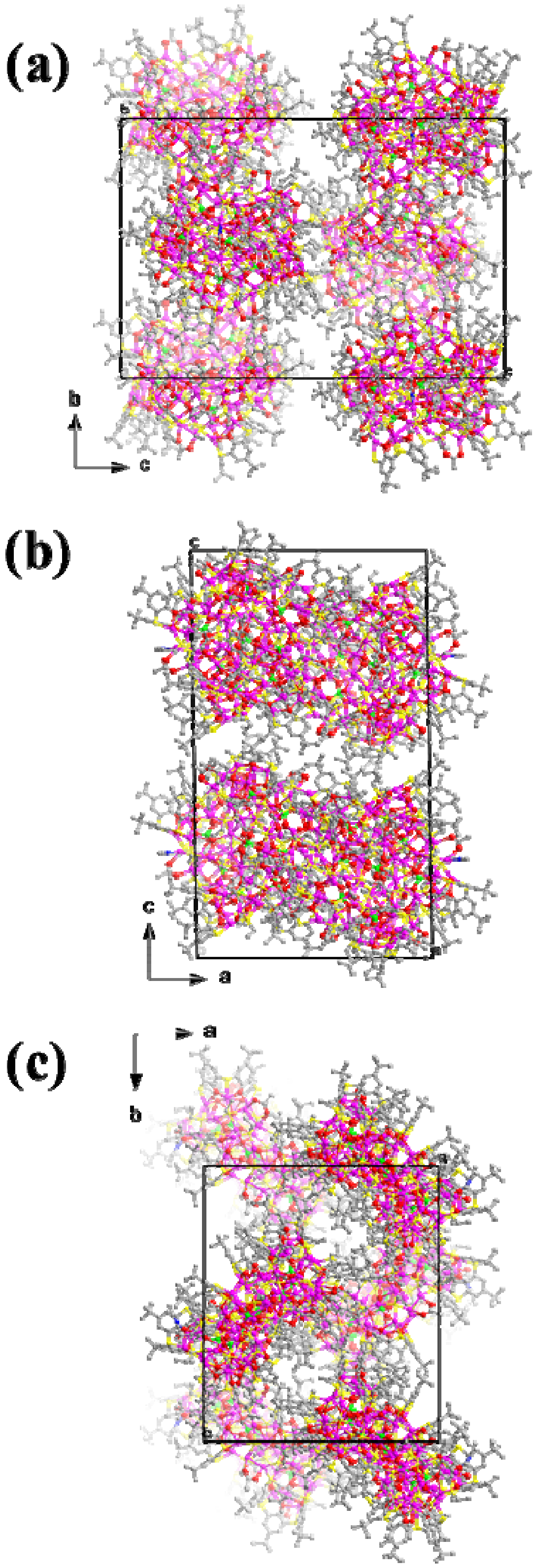

Supplementary Figure 29: Molecules packing diagrams in one unit cell of **SD/Ag88b** viewed from different directions.

Supplementary Table 1: Crystal data collection and structure refinement for **SD/Ag88a** and **SD/Ag88b**.

Compound	**SD/Ag88a**	**SD/Ag88b**
Empirical formula	$C_{428}H_{588}Ag_{88}Cr_9N_{12}O_{88}S_{64}$	$C_{418}H_{563}Ag_{88}Cr_9N_9O_{84}S_{64}$
Formula weight	19321.47	19070.14
Temperature/K	100.00(11)	99.99(10)
Crystal system	tetragonal	monoclinic
Space group	*P*4/n	$P2_1/c$
a/Å	40.6030(4)	32.5061(4)
b/Å	40.6030(4)	38.2317(7)
c/Å	23.1435(3)	56.4459(6)
α/°	90	90
β/°	90	90.9112(12)
γ/°	90	90
Volume/Å^3	38154.6(9)	70140.2(17)
Z	2	4
ρ_{calc}g/cm^3	1.682	1.806
μ/mm^{-1}	2.540	22.449
F(000)	18640.0	36728.0
Radiation	MoKα (λ = 0.71073)	CuKα (λ = 1.54184)
2Θ range for data collection/°	5.016 to 49.42	5.438 to 134.156
Index ranges	-47 ≤ h ≤ 47, -47 ≤ k ≤ 45, -27 ≤ l ≤ 26	-38 ≤ h ≤ 38, -45 ≤ k ≤ 18, -67 ≤ l ≤ 67
Reflections collected	261763	390897
Independent reflections	32406 [R_{int} = 0.0941, R_{sigma} = 0.0433]	122491 [R_{int} = 0.0981, R_{sigma} = 0.0767]
Data/parameters	32406/1479	122491/5970
Goodness-of-fit on F^2	1.130	1.057
Final R indexes [I>=2σ (I)]	R_1 = 0.0928, wR_2 = 0.2260	R_1 = 0.1141, wR_2 = 0.2774
Final R indexes [all data]	R_1 = 0.1067, wR_2 = 0.2371	R_1 = 0.1403, wR_2 = 0.2969
Largest diff. peak/hole / e Å^{-3}	2.67/-2.47	2.72/-2.88

Supplementary Table 2: Selected bond distances (Å) and angles (°) for **SD/Ag88a** and **SD/Ag88b**.

**SD/Ag88a**			
Ag1—Ag2	3.272 (3)	Ag11—Ag12	2.836 (2)
Ag1—Ag8	2.920 (3)	Ag11—S4	2.547 (5)
Ag1—Ag21^{i}	3.229 (2)	Ag11—S13	2.401 (5)
Ag1—S3^{i}	2.753 (5)	Ag11—O17	2.386 (12)
Ag1—S9^{i}	2.492 (5)	Ag11—O18	2.352 (12)
Ag1—S15	2.562 (5)	Ag12—Ag13	3.172 (3)
Ag1—O11	2.411 (19)	Ag12—Ag14	3.224 (3)
Ag2—Ag3	2.883 (3)	Ag12—Ag15	3.231 (3)
Ag2—Ag21^{i}	3.284 (2)	Ag12—S10	2.482 (6)
Ag2—S15	2.460 (5)	Ag12—O5	2.529 (15)
Ag2—S16^{i}	2.546 (5)	Ag12—O10	2.254 (13)
Ag2—O6^{i}	2.412 (14)	Ag13—Ag14	2.841 (3)
Ag2—O7	2.477 (15)	Ag13—S8^{i}	2.633 (4)
Ag3—Ag4	2.825 (2)	Ag13—S11	2.624 (6)
Ag3—Ag4^{i}	2.827 (2)	Ag13—S13	2.526 (6)
Ag3—S12	2.434 (5)	Ag13—O13	2.39 (2)
Ag3—S16^{i}	2.476 (5)	Ag14—Ag16	3.049 (3)
Ag3—O2	2.55 (3)	Ag14—Ag17	3.185 (3)
Ag4—Ag3^{ii}	2.827 (2)	Ag14—S10	2.507 (7)
Ag4—Ag5	2.901 (3)	Ag14—S11	2.472 (6)
Ag4—S12	2.487 (6)	Ag14—O14	2.22 (2)
Ag4—S16	2.459 (5)	Ag15—Ag16	3.323 (3)
Ag4—O7	2.540 (15)	Ag15—S9	2.402 (5)
Ag5—Ag12	3.310 (3)	Ag15—S10	2.402 (6)
Ag5—Ag13	3.236 (3)	Ag15—O5	2.571 (14)
Ag5—S11	2.457 (6)	Ag16—Ag19	3.225 (2)
Ag5—S12	2.538 (5)	Ag16—S6	2.511 (5)
Ag5—O3	2.455 (14)	Ag16—S10	2.397 (5)
Ag5—O10	2.467 (14)	Ag16—O20	2.443 (15)
Ag6—Ag10	3.332 (2)	Ag16—O21	2.370 (13)
Ag6—S3	2.613 (5)	Ag17—Ag19	3.135 (2)
Ag6—S16	2.571 (6)	Ag17—S7	2.597 (6)
Ag6—O5	2.471 (14)	Ag17—S11	2.452 (5)
Ag6—O10	2.492 (15)	Ag17—O19	2.450 (12)
Ag6—O15	2.417 (12)	Ag17—O20	2.474 (16)
Ag7—Ag8	3.086 (3)	Ag18—S8	2.590 (4)
Ag7—Ag10	3.133 (2)	Ag18—S12^{ii}	2.568 (6)
Ag7—S1	2.613 (5)	Ag18—O6	2.453 (14)
Ag7—S15	2.466 (5)	Ag18—O8^{ii}	2.536 (14)

Ag7—O15	2.362 (12)	Ag18—O19	2.485 (11)
Ag7—O16	2.488 (14)	Ag19—Ag20	3.104 (2)
Ag8—Ag9	3.028 (2)	Ag19—O4	2.392 (12)
Ag8—S14	2.641 (7)	Ag19—O19	2.255 (5)
Ag8—S15	2.517 (6)	Ag19—O20	2.370 (10)
Ag8—O12	2.14 (2)	Ag19—O21	2.382 (9)
Ag9—Ag10	3.202 (2)	Ag19—O22	2.377 (14)
Ag9—Ag22	3.141 (2)	Ag20—Ag21	2.871 (2)
Ag9—S2	2.540 (4)	Ag20—S5	2.521 (4)
Ag9—S14	2.425 (5)	Ag20—S9	2.384 (5)
Ag9—O16	2.419 (13)	Ag20—O21	2.393 (12)
Ag9—O17	2.333 (14)	Ag20—O22	2.377 (10)
Ag10—Ag11	3.080 (2)	Ag21—Ag22^{ii}	3.280 (2)
Ag10—O9	2.351 (14)	Ag21—S14^{ii}	2.489 (6)
Ag10—O15	2.365 (14)	Ag21—O6	2.240 (14)
Ag10—O16	2.401 (15)	Ag21—O8^{ii}	2.521 (14)
Ag10—O17	2.389 (14)	Ag22—S13	2.408 (6)
Ag10—O18	2.306 (13)	Ag22—S14	2.423 (6)
S9^{i}—Ag1—S3^{i}	90.86 (15)	O17—Ag11—S13	118.4 (4)
S9^{i}—Ag1—S15	147.48 (17)	O18—Ag11—S4	77.6 (3)
S15—Ag1—S3^{i}	102.09 (15)	O18—Ag11—S13	133.1 (3)
O11—Ag1—S3^{i}	94.3 (5)	O18—Ag11—O17	89.0 (4)
O11—Ag1—S9^{i}	104.0 (5)	S10—Ag12—O5	93.8 (3)
O11—Ag1—S15	104.6 (5)	O10—Ag12—S10	170.6 (4)
S15—Ag2—S16^{i}	123.66 (17)	O10—Ag12—O5	78.3 (5)
S15—Ag2—O7	90.0 (4)	S11—Ag13—S8^{i}	106.91 (16)
O6^{i}—Ag2—S15	124.0 (4)	S13—Ag13—S8^{i}	97.61 (15)
O6^{i}—Ag2—S16^{i}	105.1 (4)	S13—Ag13—S11	143.98 (19)
O6^{i}—Ag2—O7	93.8 (5)	O13—Ag13—S8^{i}	96.9 (5)
O7—Ag2—S16^{i}	114.4 (4)	O13—Ag13—S11	101.0 (7)
S12—Ag3—S16^{i}	164.7 (2)	O13—Ag13—S13	101.8 (7)
S12—Ag3—O2	79.5 (7)	S11—Ag14—S10	126.2 (2)
S16^{i}—Ag3—O2	114.6 (7)	O14—Ag14—S10	105.7 (7)
S12—Ag4—O7	83.9 (4)	O14—Ag14—S11	122.3 (6)
S16—Ag4—S12	164.8 (2)	S9—Ag15—O5	89.9 (4)
S16—Ag4—O7	94.2 (4)	S10—Ag15—S9	164.89 (19)
S11—Ag5—S12	126.3 (2)	S10—Ag15—O5	94.7 (4)
S11—Ag5—O10	121.7 (4)	S10—Ag16—S6	148.7 (2)
O3—Ag5—S11	89.6 (3)	S10—Ag16—O20	118.1 (3)
O3—Ag5—S12	116.1 (4)	O20—Ag16—S6	76.9 (3)
O3—Ag5—O10	90.7 (5)	O21—Ag16—S6	77.3 (3)
O10—Ag5—S12	105.3 (3)	O21—Ag16—S10	128.7 (3)
S16—Ag6—S3	112.85 (16)	O21—Ag16—O20	84.1 (3)

O5—Ag6—S3	83.9 (3)	S11—Ag17—S7	126.89 (18)
O5—Ag6—S16	90.1 (3)	S11—Ag17—O20	116.0 (3)
O5—Ag6—O10	75.2 (5)	O19—Ag17—S7	74.6 (3)
O10—Ag6—S3	144.0 (3)	O19—Ag17—S11	153.4 (3)
O10—Ag6—S16	96.3 (3)	O19—Ag17—O20	83.1 (3)
O15—Ag6—S3	74.2 (3)	O20—Ag17—S7	73.5 (3)
O15—Ag6—S16	112.3 (3)	S12^{ii}—Ag18—S8	114.92 (15)
O15—Ag6—O5	153.3 (4)	O6—Ag18—S8	138.7 (3)
O15—Ag6—O10	114.7 (4)	O6—Ag18—S12^{ii}	98.4 (3)
S15—Ag7—S1	124.68 (14)	O6—Ag18—O8^{ii}	78.0 (4)
S15—Ag7—O16	113.1 (4)	O6—Ag18—O19	113.7 (4)
O15—Ag7—S1	76.2 (3)	O8^{ii}—Ag18—S8	78.6 (3)
O15—Ag7—S15	153.1 (3)	O8^{ii}—Ag18—S12^{ii}	89.6 (3)
O15—Ag7—O16	87.4 (5)	O19—Ag18—S8	72.0 (2)
O16—Ag7—S1	74.0 (3)	O19—Ag18—S12^{ii}	118.35 (19)
S15—Ag8—S14	111.09 (19)	O19—Ag18—O8^{ii}	145.8 (4)
O12—Ag8—S14	107.6 (7)	O19—Ag19—O4	90.4 (4)
O12—Ag8—S15	126.4 (6)	O19—Ag19—O20	89.8 (5)
S14—Ag9—S2	147.87 (16)	O19—Ag19—O21	170.2 (4)
O16—Ag9—S2	75.9 (4)	O19—Ag19—O22	94.7 (4)
O16—Ag9—S14	113.5 (4)	O20—Ag19—O4	83.8 (4)
O17—Ag9—S2	77.3 (3)	O20—Ag19—O21	85.5 (5)
O17—Ag9—S14	132.2 (3)	O20—Ag19—O22	169.6 (4)
O17—Ag9—O16	86.0 (5)	O21—Ag19—O4	97.6 (4)
O9—Ag10—O15	87.8 (5)	O22—Ag19—O4	105.5 (4)
O9—Ag10—O16	83.6 (5)	O22—Ag19—O21	88.6 (4)
O9—Ag10—O17	96.9 (5)	S9—Ag20—S5	140.18 (15)
O15—Ag10—O16	89.4 (4)	S9—Ag20—O21	122.3 (3)
O15—Ag10—O17	172.4 (4)	O21—Ag20—S5	76.7 (3)
O17—Ag10—O16	85.2 (4)	O22—Ag20—S5	76.9 (2)
O18—Ag10—O9	104.1 (5)	O22—Ag20—S9	132.5 (3)
O18—Ag10—O15	94.6 (4)	O22—Ag20—O21	88.4 (4)
O18—Ag10—O16	171.4 (5)	S14^{ii}—Ag21—O8^{ii}	94.1 (4)
O18—Ag10—O17	90.0 (4)	O6—Ag21—S14^{ii}	166.3 (4)
S13—Ag11—S4	141.40 (19)	O6—Ag21—O8^{ii}	82.3 (5)
O17—Ag11—S4	77.8 (4)	S13—Ag22—S14	169.83 (19)
Symmetry codes: (i) −*y*+1/2, *x*, *z*; (ii) *y*, −*x*+1/2, *z*.			
**SD/Ag88b**			
Ag1—Ag5	3.100 (4)	Ag40—O12	2.463 (19)
Ag1—Ag38	2.902 (3)	Ag40—S37	2.480 (6)
Ag1—Ag51	2.769 (3)	Ag40—S38	2.557 (7)
Ag1—O76	2.297 (18)	Ag40—S44	2.694 (5)
Ag1—S43	2.333 (9)	Ag41—O33	2.576 (13)

Ag1—S61	2.622 (8)	Ag41—S21	2.411 (6)
Ag2—Ag31	3.087 (5)	Ag41—S50	2.419 (5)
Ag2—Ag40	3.068 (6)	Ag42—Ag53	3.370 (3)
Ag2—Ag44	2.972 (5)	Ag42—O49	2.510 (14)
Ag2—O80	2.29 (2)	Ag42—O73	2.450 (16)
Ag2—S38	2.379 (10)	Ag42—O77	2.374 (12)
Ag2—S41	2.834 (10)	Ag42—S17	2.643 (5)
Ag3—Ag17	3.075 (2)	Ag42—S28	2.577 (6)
Ag3—Ag60	3.1862 (19)	Ag43—Ag81	3.289 (2)
Ag3—Ag87	3.045 (3)	Ag43—O39	2.512 (11)
Ag3—O22	2.436 (12)	Ag43—O46	2.337 (15)
Ag3—O27	2.323 (13)	Ag43—O69	2.414 (15)
Ag3—S11	2.417 (5)	Ag43—S34	2.646 (6)
Ag3—S45	2.538 (5)	Ag43—S63	2.551 (6)
Ag4—Ag50	3.262 (2)	Ag44—Ag49	3.193 (3)
Ag4—Ag63	3.150 (2)	Ag44—O34	2.356 (19)
Ag4—O24	2.460 (13)	Ag44—O67	2.435 (17)
Ag4—O44	2.373 (12)	Ag44—S41	2.429 (7)
Ag4—S26	2.444 (5)	Ag44—S42	2.557 (7)
Ag4—S48	2.632 (5)	Ag45—Ag65	3.365 (2)
Ag5—Ag15	3.367 (2)	Ag45—Ag70	3.160 (3)
Ag5—Ag65	2.901 (2)	Ag45—S4	2.395 (7)
Ag5—O11	2.238 (13)	Ag45—S57	2.442 (7)
Ag5—O58	2.575 (12)	Ag46—Ag82	3.119 (3)
Ag5—O68	2.577 (12)	Ag46—O23	2.465 (13)
Ag5—S43	2.488 (6)	Ag46—O31	2.469 (17)
Ag6—Ag21	3.307 (2)	Ag46—S36	2.446 (6)
Ag6—Ag24	3.214 (3)	Ag46—S63	2.544 (5)
Ag6—Ag29	2.870 (3)	Ag47—Ag48	2.856 (3)
Ag6—O35	2.512 (16)	Ag47—O35	2.599 (16)
Ag6—O63	2.400 (14)	Ag47—S3	2.432 (5)
Ag6—S12	2.422 (6)	Ag47—S5	2.471 (5)
Ag6—S47	2.569 (5)	Ag48—Ag51	3.350 (3)
Ag7—Ag16	2.854 (2)	Ag48—O11	2.440 (12)
Ag7—Ag71	3.127 (2)	Ag48—O51	2.536 (14)
Ag7—O48	2.437 (12)	Ag48—S5	2.526 (5)
Ag7—O55	2.402 (13)	Ag48—S61	2.458 (7)
Ag7—S50	2.385 (5)	Ag49—Ag53	3.250 (3)
Ag7—S52	2.508 (5)	Ag49—S13	2.392 (6)
Ag8—Ag9	2.877 (2)	Ag49—S41	2.418 (7)
Ag8—Ag14	3.242 (2)	Ag50—Ag72	2.972 (2)
Ag8—O15	2.491 (14)	Ag50—O42	2.213 (16)
Ag8—O79	2.449 (12)	Ag50—S26	2.514 (5)

Ag8—S26	2.447 (5)	Ag50—S31	2.505 (7)
Ag8—S28	2.550 (5)	Ag51—O8	2.412 (18)
Ag9—Ag27	2.866 (2)	Ag51—S4	2.502 (6)
Ag9—Ag73	2.866 (2)	Ag51—S6	2.663 (4)
Ag9—O1	2.58 (3)	Ag51—S61	2.561 (6)
Ag9—S9	2.418 (5)	Ag52—O71	2.518 (13)
Ag9—S28	2.450 (5)	Ag52—O73	2.427 (15)
Ag10—Ag54	3.171 (3)	Ag52—S38	2.424 (8)
Ag10—Ag57	3.001 (3)	Ag52—S54	2.535 (5)
Ag10—Ag68	3.234 (2)	Ag53—Ag78	2.949 (3)
Ag10—O19	2.438 (13)	Ag53—O18	2.511 (13)
Ag10—O32	2.336 (13)	Ag53—O49	2.586 (16)
Ag10—S51	2.553 (4)	Ag53—O73	2.334 (17)
Ag10—S56	2.406 (5)	Ag53—S41	2.428 (8)
Ag11—Ag19	3.352 (2)	Ag54—S32	2.425 (6)
Ag11—O41	2.450 (14)	Ag54—S56	2.452 (8)
Ag11—O70	2.556 (12)	Ag55—Ag72	3.301 (3)
Ag11—O79	2.482 (14)	Ag55—S27	2.427 (7)
Ag11—S9	2.592 (5)	Ag55—S31	2.456 (7)
Ag11—S49	2.619 (5)	Ag56—Ag57	2.907 (3)
Ag12—Ag36	3.125 (2)	Ag56—Ag84	3.291 (3)
Ag12—Ag73	2.887 (2)	Ag56—O61	2.56 (3)
Ag12—O43	2.478 (12)	Ag56—S22	2.549 (6)
Ag12—O83	2.442 (12)	Ag56—S35	2.476 (6)
Ag12—S9	2.539 (5)	Ag56—S46	2.675 (5)
Ag12—S58	2.484 (6)	Ag57—Ag81	3.376 (4)
Ag13—Ag71	3.380 (2)	Ag57—O56	2.34 (3)
Ag13—O10	2.450 (12)	Ag57—S22	2.497 (7)
Ag13—O14	2.542 (15)	Ag57—S56	2.547 (9)
Ag13—O40	2.495 (11)	Ag58—Ag82	2.868 (3)
Ag13—S14	2.639 (5)	Ag58—O7	2.24 (2)
Ag13—S47	2.558 (5)	Ag58—S30	2.516 (7)
Ag14—Ag19	3.355 (3)	Ag58—S36	2.505 (7)
Ag14—Ag50	2.873 (2)	Ag59—Ag83	3.302 (3)
Ag14—O9	2.465 (15)	Ag59—S35	2.397 (6)
Ag14—S13	2.499 (6)	Ag59—S43	2.391 (7)
Ag14—S17	2.691 (5)	Ag60—Ag62	3.134 (2)
Ag14—S26	2.601 (5)	Ag60—Ag67	3.202 (2)
Ag15—O11	2.500 (13)	Ag60—O22	2.394 (11)
Ag15—O45	2.359 (15)	Ag60—O27	2.482 (12)
Ag15—O68	2.509 (14)	Ag60—O50	2.431 (13)
Ag15—S3	2.574 (6)	Ag60—O52	2.431 (14)
Ag15—S46	2.640 (5)	Ag60—O57	2.304 (12)

Ag16—Ag17	3.271 (2)	Ag61—Ag65	3.118 (2)
Ag16—Ag77	3.192 (2)	Ag61—Ag70	3.255 (2)
Ag16—Ag79	3.321 (2)	Ag61—Ag74	3.121 (2)
Ag16—O14	2.280 (12)	Ag61—O16	2.418 (15)
Ag16—O40	2.521 (12)	Ag61—O37	2.418 (11)
Ag16—S11	2.492 (5)	Ag61—O45	2.402 (17)
Ag17—S11	2.449 (6)	Ag61—O53	2.444 (16)
Ag17—S15	2.406 (5)	Ag61—O58	2.366 (14)
Ag18—Ag34	3.155 (2)	Ag62—O27	2.383 (12)
Ag18—O28	2.48 (2)	Ag62—O57	2.381 (13)
Ag18—O46	2.366 (14)	Ag62—S8	2.538 (5)
Ag18—S61	2.442 (5)	Ag62—S15	2.404 (5)
Ag18—S64	2.622 (6)	Ag63—Ag69	3.367 (2)
Ag19—Ag39	2.909 (2)	Ag63—Ag72	3.226 (2)
Ag19—Ag50	3.351 (3)	Ag63—O6	2.310 (14)
Ag19—O29	2.592 (14)	Ag63—O24	2.379 (15)
Ag19—O70	2.506 (12)	Ag63—O26	2.399 (13)
Ag19—O79	2.291 (13)	Ag63—O44	2.374 (14)
Ag19—S31	2.484 (5)	Ag63—O78	2.470 (14)
Ag20—Ag28	3.232 (2)	Ag64—Ag68	3.3722 (19)
Ag20—Ag78	3.136 (2)	Ag64—O5	2.385 (12)
Ag20—Ag85	3.175 (2)	Ag64—O23	2.476 (16)
Ag20—O18	2.279 (14)	Ag64—O60	2.501 (12)
Ag20—O21	2.307 (14)	Ag64—S44	2.658 (5)
Ag20—O54	2.507 (14)	Ag64—S54	2.573 (5)
Ag20—O62	2.378 (16)	Ag65—O16	2.431 (13)
Ag20—O77	2.463 (13)	Ag65—O58	2.401 (12)
Ag21—Ag45	3.161 (3)	Ag65—S4	2.388 (5)
Ag21—Ag62	2.881 (2)	Ag65—S19	2.533 (5)
Ag21—Ag66	3.376 (3)	Ag66—O52	2.371 (12)
Ag21—O57	2.561 (11)	Ag66—O63	2.441 (16)
Ag21—O63	2.299 (14)	Ag66—O65	2.544 (13)
Ag21—O65	2.539 (15)	Ag66—S5	2.572 (5)
Ag21—S57	2.440 (6)	Ag66—S6	2.625 (4)
Ag22—Ag41	3.248 (2)	Ag67—Ag87	3.150 (3)
Ag22—Ag71	3.200 (2)	Ag67—O22	2.454 (12)
Ag22—Ag86	3.222 (2)	Ag67—O52	2.443 (12)
Ag22—O17	2.411 (12)	Ag67—S7	2.596 (5)
Ag22—O55	2.353 (14)	Ag67—S16	2.488 (5)
Ag22—S20	2.568 (5)	Ag68—O5	2.467 (14)
Ag22—S21	2.431 (6)	Ag68—O19	2.306 (13)
Ag23—Ag35	2.890 (2)	Ag68—O30	2.284 (14)
Ag23—Ag68	3.110 (2)	Ag68—O32	2.541 (14)

Ag23—O30	2.428 (15)	Ag68—O66	2.430 (13)
Ag23—O32	2.345 (13)	Ag69—O33	2.502 (11)
Ag23—S29	2.514 (5)	Ag69—O44	2.402 (13)
Ag23—S32	2.402 (5)	Ag69—O83	2.453 (12)
Ag24—Ag88	2.896 (3)	Ag69—S10	2.647 (5)
Ag24—O36	2.433 (17)	Ag69—S18	2.568 (5)
Ag24—S12	2.604 (6)	Ag70—Ag88	2.908 (3)
Ag24—S14	2.707 (5)	Ag70—O16	2.308 (13)
Ag24—S15	2.491 (5)	Ag70—O53	2.446 (17)
Ag25—Ag37	2.875 (2)	Ag70—S55	2.536 (5)
Ag25—Ag47	2.851 (2)	Ag70—S57	2.408 (5)
Ag25—Ag84	2.884 (2)	Ag71—Ag76	3.161 (2)
Ag25—S3	2.477 (5)	Ag71—O10	2.396 (12)
Ag25—S63	2.457 (5)	Ag71—O13	2.418 (15)
Ag26—Ag31	3.116 (2)	Ag71—O17	2.460 (13)
Ag26—Ag39	3.127 (2)	Ag71—O48	2.346 (13)
Ag26—Ag44	3.226 (2)	Ag71—O55	2.403 (13)
Ag26—O29	2.372 (12)	Ag72—O24	2.408 (16)
Ag26—O34	2.469 (17)	Ag72—O78	2.413 (14)
Ag26—O41	2.401 (15)	Ag72—S1	2.555 (5)
Ag26—O67	2.459 (16)	Ag72—S31	2.426 (5)
Ag26—O82	2.412 (15)	Ag73—Ag75	2.859 (2)
Ag27—Ag37	2.845 (3)	Ag73—O4	2.58 (3)
Ag27—Ag52	2.880 (3)	Ag73—O15	2.577 (14)
Ag27—S28	2.440 (5)	Ag73—S9	2.482 (5)
Ag27—S54	2.464 (4)	Ag73—S18	2.463 (5)
Ag28—Ag33	3.306 (3)	Ag74—Ag88	3.254 (3)
Ag28—Ag58	2.952 (3)	Ag74—O45	2.417 (16)
Ag28—O21	2.440 (12)	Ag74—O53	2.475 (16)
Ag28—O54	2.360 (15)	Ag74—S12	2.454 (5)
Ag28—S25	2.547 (5)	Ag74—S59	2.614 (6)
Ag28—S30	2.424 (5)	Ag75—Ag79	2.910 (2)
Ag29—Ag47	2.862 (2)	Ag75—O43	2.564 (13)
Ag29—Ag75	2.847 (2)	Ag75—S18	2.466 (5)
Ag29—O3	2.52 (4)	Ag75—S47	2.446 (5)
Ag29—S5	2.449 (5)	Ag76—Ag86	3.126 (2)
Ag29—S47	2.467 (5)	Ag76—O10	2.374 (13)
Ag30—Ag63	3.099 (2)	Ag76—O17	2.413 (11)
Ag30—Ag80	2.901 (2)	Ag76—S24	2.596 (5)
Ag30—O6	2.514 (15)	Ag76—S58	2.440 (5)
Ag30—O78	2.367 (14)	Ag77—Ag79	3.121 (2)
Ag30—S27	2.402 (6)	Ag77—Ag87	3.035 (3)
Ag30—S60	2.532 (6)	Ag77—O75	2.446 (17)

Ag31—O41	2.349 (15)	Ag77—S10	2.822 (5)
Ag31—O67	2.470 (18)	Ag77—S16	2.535 (5)
Ag31—S38	2.448 (6)	Ag77—S50	2.465 (5)
Ag31—S40	2.611 (6)	Ag78—O18	2.424 (12)
Ag32—Ag57	3.141 (3)	Ag78—O54	2.387 (13)
Ag32—Ag68	3.181 (2)	Ag78—S37	2.398 (5)
Ag32—O5	2.348 (13)	Ag78—S53	2.534 (5)
Ag32—O19	2.457 (12)	Ag79—O14	2.426 (14)
Ag32—S22	2.455 (5)	Ag79—O20	2.464 (13)
Ag32—S23	2.636 (5)	Ag79—S16	2.445 (5)
Ag33—Ag35	3.377 (3)	Ag79—S18	2.545 (5)
Ag33—Ag78	3.347 (2)	Ag80—Ag86	3.054 (2)
Ag33—O60	2.598 (12)	Ag80—O33	2.577 (12)
Ag33—S30	2.393 (7)	Ag80—O83	2.287 (14)
Ag33—S37	2.400 (7)	Ag80—S21	2.559 (6)
Ag34—Ag38	3.214 (2)	Ag81—Ag83	2.932 (3)
Ag34—Ag83	3.105 (2)	Ag81—O38	2.561 (16)
Ag34—O25	2.513 (18)	Ag81—O39	2.533 (15)
Ag34—O28	2.306 (16)	Ag81—O69	2.234 (14)
Ag34—O38	2.251 (14)	Ag81—S56	2.459 (5)
Ag34—O46	2.464 (16)	Ag82—O81	2.495 (19)
Ag34—O72	2.394 (15)	Ag82—S32	2.464 (5)
Ag35—Ag58	3.212 (3)	Ag82—S34	2.670 (6)
Ag35—Ag82	3.206 (3)	Ag82—S36	2.556 (6)
Ag35—O23	2.234 (15)	Ag83—O25	2.378 (18)
Ag35—S30	2.493 (6)	Ag83—O38	2.416 (15)
Ag36—Ag80	3.164 (3)	Ag83—S35	2.423 (6)
Ag36—Ag86	2.873 (3)	Ag83—S62	2.544 (7)
Ag36—O74	2.408 (16)	Ag84—O47	2.449 (13)
Ag36—S27	2.471 (6)	Ag84—O69	2.472 (14)
Ag36—S49	2.742 (5)	Ag84—S3	2.536 (5)
Ag36—S58	2.561 (6)	Ag84—S22	2.482 (5)
Ag37—Ag46	2.899 (3)	Ag85—O21	2.471 (13)
Ag37—O47	2.558 (15)	Ag85—O77	2.303 (14)
Ag37—S54	2.444 (5)	Ag85—S33	2.614 (5)
Ag37—S63	2.455 (6)	Ag85—S36	2.440 (6)
Ag38—O25	2.358 (16)	Ag86—O59	2.254 (19)
Ag38—O28	2.41 (2)	Ag86—S21	2.487 (6)
Ag38—S39	2.552 (6)	Ag86—S58	2.534 (6)
Ag38—S43	2.466 (6)	Ag87—N1	2.45 (3)
Ag39—Ag49	3.349 (3)	Ag87—O84	2.33 (2)
Ag39—O29	2.377 (14)	Ag87—S11	2.544 (7)
Ag39—O34	2.427 (16)	Ag87—S16	2.628 (6)

Ag39—S2	2.546 (6)	Ag88—O64	2.247 (19)
Ag39—S13	2.410 (5)	Ag88—S12	2.502 (7)
Ag40—Ag52	3.256 (3)	Ag88—S57	2.604 (8)
O76—Ag1—S43	123.7 (6)	S41—Ag44—O67	114.5 (5)
O76—Ag1—S61	109.7 (6)	S41—Ag44—S42	149.0 (2)
S43—Ag1—S61	126.6 (2)	S4—Ag45—S57	167.4 (2)
O80—Ag2—S38	123.5 (6)	O23—Ag46—O31	91.0 (5)
O80—Ag2—S41	104.8 (7)	O23—Ag46—S63	108.3 (4)
S38—Ag2—S41	109.6 (3)	O31—Ag46—S63	113.2 (4)
O22—Ag3—S45	77.1 (3)	S36—Ag46—O23	117.3 (4)
O27—Ag3—O22	88.4 (4)	S36—Ag46—O31	89.4 (4)
O27—Ag3—S11	135.8 (3)	S36—Ag46—S63	128.49 (19)
O27—Ag3—S45	76.6 (3)	S3—Ag47—O35	98.1 (4)
S11—Ag3—O22	111.6 (3)	S3—Ag47—S5	164.97 (19)
S11—Ag3—S45	144.51 (16)	S5—Ag47—O35	81.5 (4)
O24—Ag4—S48	73.9 (4)	O11—Ag48—O51	92.3 (4)
O44—Ag4—O24	86.1 (5)	O11—Ag48—S5	107.9 (3)
O44—Ag4—S26	155.5 (3)	O11—Ag48—S61	114.0 (4)
O44—Ag4—S48	75.7 (3)	S5—Ag48—Ag51	117.82 (14)
S26—Ag4—O24	113.8 (4)	S5—Ag48—O51	119.1 (4)
S26—Ag4—S48	122.16 (17)	S61—Ag48—O51	87.7 (3)
O11—Ag5—O58	86.9 (4)	S61—Ag48—S5	128.70 (18)
O11—Ag5—O68	84.8 (5)	S13—Ag49—S41	171.9 (2)
O11—Ag5—S43	168.2 (4)	O42—Ag50—S26	111.5 (5)
O58—Ag5—O68	82.1 (4)	O42—Ag50—S31	120.6 (6)
S43—Ag5—O58	104.3 (3)	S31—Ag50—S26	124.68 (18)
S43—Ag5—O68	92.9 (3)	O8—Ag51—S4	104.8 (6)
O35—Ag6—S47	113.5 (4)	O8—Ag51—S6	93.8 (4)
O63—Ag6—O35	93.9 (5)	O8—Ag51—S61	99.3 (6)
O63—Ag6—S12	128.2 (4)	S4—Ag51—S6	97.29 (18)
O63—Ag6—S47	105.0 (3)	S4—Ag51—S61	145.67 (18)
S12—Ag6—O35	90.3 (4)	S61—Ag51—S6	105.17 (18)
S12—Ag6—S47	120.14 (18)	O71—Ag52—S54	111.8 (4)
O48—Ag7—S52	79.4 (3)	O73—Ag52—O71	94.6 (5)
O55—Ag7—O48	89.6 (4)	O73—Ag52—S54	103.5 (4)
O55—Ag7—S52	78.4 (3)	S38—Ag52—O71	92.9 (3)
S50—Ag7—O48	129.1 (3)	S38—Ag52—O73	123.1 (5)
S50—Ag7—O55	119.7 (4)	S38—Ag52—S54	125.2 (2)
S50—Ag7—S52	142.46 (17)	O18—Ag53—O49	86.3 (4)
O15—Ag8—S28	119.4 (3)	O73—Ag53—O18	88.4 (5)
O79—Ag8—O15	91.3 (5)	O73—Ag53—O49	82.3 (5)
O79—Ag8—S28	103.1 (3)	O73—Ag53—S41	162.0 (4)
S26—Ag8—O15	88.8 (3)	S41—Ag53—O18	109.4 (4)

S26—Ag8—O79	118.6 (4)	S41—Ag53—O49	96.0 (4)
S26—Ag8—S28	129.25 (17)	S32—Ag54—S56	160.2 (3)
S9—Ag9—O1	92.5 (7)	S27—Ag55—S31	158.9 (2)
S9—Ag9—S28	166.17 (19)	O61—Ag56—S46	91.4 (6)
S28—Ag9—O1	101.2 (7)	S22—Ag56—O61	97.6 (7)
O19—Ag10—S51	75.5 (3)	S22—Ag56—S46	108.42 (19)
O32—Ag10—O19	85.9 (5)	S35—Ag56—O61	100.2 (7)
O32—Ag10—S51	78.5 (3)	S35—Ag56—S22	143.0 (2)
O32—Ag10—S56	130.3 (4)	S35—Ag56—S46	103.26 (19)
S56—Ag10—O19	122.1 (4)	O56—Ag57—S22	112.7 (8)
S56—Ag10—S51	143.36 (18)	O56—Ag57—S56	116.1 (8)
O41—Ag11—O70	146.5 (5)	S22—Ag57—S56	126.3 (2)
O41—Ag11—O79	113.7 (4)	O7—Ag58—S30	120.6 (7)
O41—Ag11—S9	114.3 (4)	O7—Ag58—S36	111.3 (7)
O41—Ag11—S49	72.9 (4)	S36—Ag58—S30	126.6 (2)
O70—Ag11—S9	91.1 (3)	S43—Ag59—S35	168.4 (3)
O70—Ag11—S49	77.6 (3)	O22—Ag60—O27	85.8 (4)
O79—Ag11—O70	80.7 (4)	O22—Ag60—O50	78.3 (4)
O79—Ag11—S9	100.0 (3)	O22—Ag60—O52	89.4 (4)
O79—Ag11—S49	141.7 (3)	O50—Ag60—O27	100.5 (5)
S9—Ag11—S49	111.60 (17)	O52—Ag60—O27	172.5 (4)
O43—Ag12—S9	120.7 (3)	O52—Ag60—O50	84.1 (5)
O43—Ag12—S58	84.7 (3)	O57—Ag60—O22	172.4 (4)
O83—Ag12—O43	90.8 (4)	O57—Ag60—O27	89.2 (4)
O83—Ag12—S9	107.5 (3)	O57—Ag60—O50	108.3 (4)
O83—Ag12—S58	116.3 (3)	O57—Ag60—O52	94.9 (4)
S58—Ag12—S9	128.95 (17)	O16—Ag61—O37	93.9 (4)
O10—Ag13—O14	112.8 (4)	O16—Ag61—O53	84.3 (5)
O10—Ag13—O40	151.8 (4)	O37—Ag61—O53	82.0 (4)
O10—Ag13—S14	73.5 (3)	O45—Ag61—O16	174.3 (5)
O10—Ag13—S47	113.4 (3)	O45—Ag61—O37	89.2 (5)
O14—Ag13—S14	142.0 (3)	O45—Ag61—O53	91.4 (5)
O14—Ag13—S47	96.5 (3)	O58—Ag61—O16	91.4 (5)
O40—Ag13—O14	78.8 (4)	O58—Ag61—O37	102.2 (4)
O40—Ag13—S14	82.0 (3)	O58—Ag61—O45	92.7 (5)
O40—Ag13—S47	89.5 (3)	O58—Ag61—O53	174.2 (5)
S47—Ag13—S14	115.77 (15)	O27—Ag62—S8	77.3 (3)
O9—Ag14—S13	104.5 (5)	O27—Ag62—S15	118.0 (3)
O9—Ag14—S17	93.0 (3)	O57—Ag62—O27	89.8 (4)
O9—Ag14—S26	96.5 (5)	O57—Ag62—S8	78.2 (3)
S13—Ag14—S17	98.64 (17)	O57—Ag62—S15	128.7 (3)
S13—Ag14—S26	144.79 (18)	S15—Ag62—S8	145.79 (18)
S26—Ag14—S17	108.27 (16)	O6—Ag63—O24	171.9 (5)

O11—Ag15—O68	81.1 (4)	O6—Ag63—O26	106.4 (5)
O11—Ag15—S3	101.2 (3)	O6—Ag63—O44	93.1 (5)
O11—Ag15—S46	136.7 (3)	O6—Ag63—O78	92.6 (5)
O45—Ag15—O11	113.5 (5)	O24—Ag63—O26	81.7 (5)
O45—Ag15—O68	147.1 (5)	O24—Ag63—O78	85.5 (5)
O45—Ag15—S3	112.9 (4)	O26—Ag63—O78	97.8 (5)
O45—Ag15—S46	73.5 (4)	O44—Ag63—O24	88.0 (5)
O68—Ag15—S3	91.4 (3)	O44—Ag63—O26	87.6 (5)
O68—Ag15—S46	76.1 (3)	O44—Ag63—O78	170.8 (4)
S3—Ag15—S46	115.40 (16)	O5—Ag64—O23	114.6 (5)
O14—Ag16—O40	83.3 (5)	O5—Ag64—O60	150.9 (4)
O14—Ag16—S11	167.6 (4)	O5—Ag64—S44	74.3 (3)
S11—Ag16—O40	94.4 (3)	O5—Ag64—S54	111.1 (3)
S15—Ag17—S11	170.93 (18)	O23—Ag64—O60	78.4 (5)
O28—Ag18—S64	74.8 (4)	O23—Ag64—S44	138.7 (4)
O46—Ag18—O28	87.4 (6)	O23—Ag64—S54	100.2 (3)
O46—Ag18—S61	159.3 (4)	O60—Ag64—S44	79.2 (3)
O46—Ag18—S64	76.6 (3)	O60—Ag64—S54	90.6 (3)
S61—Ag18—O28	108.9 (5)	S54—Ag64—S44	114.28 (17)
S61—Ag18—S64	119.28 (18)	O16—Ag65—S19	76.6 (3)
O70—Ag19—O29	81.3 (4)	O58—Ag65—O16	90.2 (5)
O79—Ag19—O29	88.1 (4)	O58—Ag65—S19	78.1 (3)
O79—Ag19—O70	85.6 (5)	S4—Ag65—O16	119.8 (4)
O79—Ag19—S31	167.5 (3)	S4—Ag65—O58	132.1 (3)
S31—Ag19—O29	104.4 (3)	S4—Ag65—S19	140.90 (18)
S31—Ag19—O70	94.8 (3)	O52—Ag66—O63	111.9 (4)
O18—Ag20—O21	174.0 (5)	O52—Ag66—O65	148.3 (5)
O18—Ag20—O54	91.7 (5)	O52—Ag66—S5	117.2 (4)
O18—Ag20—O62	104.9 (5)	O52—Ag66—S6	73.8 (3)
O18—Ag20—O77	96.2 (5)	O63—Ag66—O65	79.9 (5)
O21—Ag20—O54	85.3 (5)	O63—Ag66—S5	98.3 (4)
O21—Ag20—O62	80.5 (5)	O63—Ag66—S6	137.4 (4)
O21—Ag20—O77	86.7 (4)	O65—Ag66—S5	88.5 (4)
O62—Ag20—O54	94.8 (5)	O65—Ag66—S6	78.0 (3)
O62—Ag20—O77	84.2 (5)	S5—Ag66—S6	116.93 (16)
O77—Ag20—O54	172.0 (5)	O22—Ag67—S7	74.2 (3)
O63—Ag21—O57	87.8 (5)	O22—Ag67—S16	114.6 (3)
O63—Ag21—O65	82.7 (5)	O52—Ag67—O22	87.7 (4)

Supplementary Table 3: The assigned formulae of species found in ESI-MS of **SD/Ag88a** dissolved in CH_2Cl_2.

	Species	Exp.	Sim.
**1a**	$[(CrO_4)_4@Ag_{42}(TC4A)_4(EtS)_{14}(OAc)(CH_2Cl_2)]^{3+}$	2953.884	2953.887
**1b**	$[(CrO_4)_4@Ag_{43}(TC4A)_4(EtS)_{16}]^{3+}$	2982.523	2982.541
**1c**	$[(CrO_4)_4@Ag_{43}(TC4A)_4(EtS)_{14}(OAc)_2(CH_2Cl_2)]^{3+}$	3009.850	3009.859
**1d**	$[(CrO_4)_4@Ag_{44}(TC4A)_4(EtS)_{16}(OAc)]^{3+}$	3038.487	3038.513
**1e**	$[(CrO_4)_4@Ag_{45}(TC4A)_4(EtS)_{16}(OAc)_2]^{3+}$	3093.784	3093.819
**1f**	$[(CrO_4)_4@Ag_{43}(TC4A)_4(EtS)_{16}(OAc)]^{2+}$	4503.247	4503.318
**1g**	$[(CrO_4)_4@Ag_{44}(TC4A)_4(EtS)_{16}(OAc)_2]^{2+}$	4587.207	4587.277

Supplementary References

1) Wang, Z. *et al.* Enclosing classical polyoxometallates in silver nanoclusters. *Nanoscale* **11**, 10927-10931 (2019).

2) Iki, N. *et al.* Synthesis of p-tert-butylthiacalix 4 arene and its inclusion property. *Tetrahedron* **56**, 1437-1443 (2000).

3) Rigaku Oxford Diffraction. *CrysAlis^{Pro} Software system, version 1.171.40.25a*, Rigaku Corporation: Oxford, UK, (2018).

4) Palatinus, L. & Chapuis, G. SUPERFLIP - a computer program for the solution of crystal structures by charge flipping in arbitrary dimensions. *J Appl Crystallogr* **40**, 786-790 (2007).

5) Sheldrick, G.M. Crystal structure refinement with SHELXL. *Acta Crystallographica Section C-Structural Chemistry* **71**, 3-8 (2015).

6) Dolomanov, O.V., Bourhis, L.J., Gildea, R.J., Howard, J.A.K. & Puschmann, H. OLEX2: a complete structure solution, refinement and analysis program. *J Appl Crystallogr* **42**, 339-341 (2009).

7) Spek, A.L. Structure validation in chemical crystallography. *Acta Crystallogr D* **65**, 148-155 (2009).

8) Voss, N.R. & Gerstein, M. 3V: cavity, channel and cleft volume calculator and extractor. *Nucleic Acids Res* **38**, W555-W562 (2010).

9) Turner, M. *et al.* CrystalExplorer17. University of Western Australia, Nedlands, Western Australia. http://hirshfeldsurface.net. (2017).
